# Spatial Expression Analysis of Odorant Binding Proteins in Both Sexes of the Aphid Parasitoid *Aphidius gifuensis* and Their Ligand Binding Properties

**DOI:** 10.3389/fphys.2022.877133

**Published:** 2022-05-04

**Authors:** Xin Jiang, Yaoguo Qin, Jun Jiang, Yun Xu, Frédéric Francis, Jia Fan, Julian Chen

**Affiliations:** ^1^ State Key Laboratory for Biology of Plant Diseases and Insect Pests, Institute of Plant Protection, Chinese Academy of Agricultural Sciences, Beijing, China; ^2^ Functional and Evolutionary Entomology, Gembloux Agro-Bio Tech, University of Liège, Gembloux, Belgium; ^3^ Department of Entomology and MOA Key Laboratory for Monitoring and Environment-Friendly Control of Crop Pests, College of Plant Protection, China Agricultural University, Beijing, China; ^4^ Agricultural Environment and Resources Institute of YAAS, Kunming, China

**Keywords:** *Aphidifus gifuensis*, transcriptome, odorant binding protein, spatial expression pattern, fluorescence binding assay

## Abstract

In China, *Aphidius gifuensis* is one of the most common endoparasitoids of the green peach aphid *Myzus persicae* and grain aphid *Sitobion miscanthi* in the field. Insect odorant-binding proteins (OBPs) play vital roles in odor perception during feeding, host searching, mating and oviposition. In addition, some OBPs are involved in other physiological processes such as gustation and reproduction. In the present study, a comparative antennal transcriptomic analysis was applied between male and female *A. gifuensis*. The spatial expression patterns among antennae, heads, thoraxes, abdomens and legs of OBPs in both sexes were further profiled. Fifteen *AgifOBP*s were predicted, and 14 of them were identified by gene cloning, including 12 classic OBPs and 2 min-C OBPs. As expected, all OBPs were mainly expressed at high levels in antennae, heads or legs which are sensory organs and tissues. Finally, ligand binding properties of 2 OBPs (AgifOBP7 and AgifOBP9) were further evaluated. Female leg specifically expressed AgifOBP9 displays a broad and high binding property to aphid alarm pheromones, plant green volatiles and aphid sex pheromones (Ki < 10 μΜ). However, female leg specifically expressed AgifOBP7 displays poor affinity for all tested ligands except CAU-II-11 ((E)-3,7-dimethylocta-2,6-dien-1-yl-2-hydroxy-3-methoxybenzoate), a reported (E)-β-farnesene (EBF) analog with an exceptionally high binding affinity (Ki = 1.07 ± 0.08 μΜ). In summary, we reported the spatial expression pattern of the OBP repertoire in *A. gifuensis*, and further studied the binding properties of OBP7 and OBP9, which are mainly expressed in female legs, laying the foundation for the dissection of the contribution of OBPs to chemosensation in *A. gifuensis*.

## Introduction

*Aphidius gifuensis* is one of the most common endoparasitoids of green peach aphid *Myzus persicae* and grain aphid *Sitobion miscanthi* in China. *S. miscanthi* is also habitually called *Sitobion avenae* in China (Zhang, 1999; Jiang et al., 2019), and is the undisputed dominant Chinese dominant pest of wheat. Aphids has long been the most damaging pest of crops and vegetables, causing yield and quality losses by stealing nutrients, transferring plant viruses, and excreting honeydew to block plant photosynthesis (Wu, 2002). In Yunnan and many other areas of China, *M. persicae* on tobacco has been successfully controlled by artificially released *A. gifuensis* as a powerful biocontrol tool (Ohta and Honda, 2010; Yang et al., 2009).

The behavioral response of insects to olfactory cues is essentially driven by feeding, reproduction and habitat selection (Pelosi et al., 2014). Molecular odorants enter the sensilla through pores and spread inside the hemolymph on the antennae due to odorant-binding proteins (OBPs) and/or chemosensory proteins (CSPs) (Pelosi et al., 2006; Leal, 2013). These odorants are then transported to olfactory receptors (ORs), ionotropic receptors (IRs), or sensory neuron membrane proteins (SNMPs), from which the chemical signals will be transmitted into electrophysiological signals for the brain (Leal, 2013; Pelosi et al., 2018). Insect OBPs were initially discovered in antennae of the moth *Antheraea polyphemus* (Vogt and Riddiford, 1981). Their wide distributions in antennal sensilla indicated the first link of OBPs in the signal chain of odorant perception (Xu et al., 2009). OBPs are tiny, globular, water-soluble proteins with a molecular weight of 10–30 kDa (Pelosi et al., 2005). The presence of six highly conserved cysteine residues, which are paired in three interlocking disulfide bridges to maintain the protein’s tertiary structure, is a common feature of classical OBPs (Pelosi et al., 2014). OBPs act as shuttles for hydrophobic odor molecules, transporting them through the sensillum lymph to odorant receptors (Zhou et al., 2010). After initiating receptors, OBPs may also concentrate odorants in the sensillum lymph and swiftly destroy odorant molecules (Vogt and Riddiford, 1981; Leal, 2013). The prediction of the whole OBP family in species became quite simple due to the availability of more insect genomes and transcriptomes using next-generation sequencing techniques. However, the number of OBPs in Hymenoptera varies; for example, *Apis mellifera* has 21 OBPs, *Microplitis mediator* has 18 OBPs, *Pieris rapae* has 14 OBPs, *Spodoptera exigua* has 34 OBPs, *Cotesia vestalis* has 20 OBPs, and 90 OBPs were predicted in *Nasonia vitripennis* (Forêt and Maleszka, 2006; Vieira et al., 2012; Liu et al., 2015; Peng et al., 2017; Li et al., 2020; Liu et al., 2020; Zhou et al., 2021). Insect OBPs not only are expressed in the chemosensory system, but also occur in nonsensory tissues and organs, such as the cornicles (Wang et al., 2021a), thoraxes (Xue et al., 2016; Gao et al., 2018; Wang et al., 2019), reproductive organs (Li et al., 2008; Sun et al., 2012b), mandibular glands (Iovinella et al., 2011), salivary glands (Zhang et al., 2017), and wings (Calvello et al., 2003; Pelosi et al., 2005; Wang et al., 2021b). Some insect OBPs have physiological functions other than binding odorants. For example, the sperm carrier function of OBPs has been reported in the male reproductive apparatus of mosquitoes (Li et al., 2008). Moreover, one OBP expressed by male moths is found on the surface of fertilized eggs, which functions to avoid cannibalistic behaviors among larvae (Sun et al., 2012b). Therefore, spatial expression patterns would be helpful to classify and analyze the possible functions of OBPs.

Herbivore-induced volatiles (HIPVs), green leaf volatiles (GLVs), and pheromones such as the aphid alarm pheromone E-beta-farnesene (EBF) are used by natural enemies to find their prey during predation and parasitism (Dong et al., 2008; CMD De Moraes et al., 1998; Buitenhuis et al., 2004). *A. gifuensis* evolved a comprehensive chemosensory system to effectively detect the semiochemical cues of its host and plants (Yang et al., 2009). For example, *A. gifuensis* can distinguish healthy, mechanically damaged, and aphid-infested plants (Dong et al., 2008). Additionally, both female and male *A. gifuensis* were reported to present a positive electroantennogram (EAG) response to EBF and many tobacco volatiles, including trans-2-hexenal, methyl salicylate, benzaldehyde, cis-3-hexen-1-ol, and 1-hexanal (Song et al., 2021a). The volatile sex pheromone has also been shown to be released by female *Aphidius*, causing intense sexual orientation in males (Fan et al., 2018). OBPs, CSPs and chemosensory receptors in *A. gifuensis* have been widely predicted based on transcriptome data (Kang et al., 2017; Fan et al., 2018). However, there is still a paucity of information on the expression profiles of odorant binding proteins in various sensory organs of *A. gifuensis.* Sequence identification is critical for further functional studies, not to mention the mechanisms of host foraging and mating behavior which are completely unknown.

In the present study, we performed gene prediction, identification, expression profiling of *AgifOBPs* and further performed a ligand competitive binding test on their recombinant proteins expressed in a prokaryotic expression system to discover two leg-specifically expressed OBPs (AgifOBP7 and AgifOBP9) in *A. gifuensis* as follows: 1) used *A. gifuensis* antennal transcriptome to predict *AgifOBPs*; 2) we identified *AgifOBPs* and profiled their spatial expression patterns among tissues and organs of both sexes; and 3) we revealed a partial mechanism of olfactory perception based on the ligand competitive binding test.

## Materials and Methods

### Insect Rearing and Tissue Collection

The laboratory population of *Aphidius gifuensis* was the same as that previously described by Fan et al., 2018. The mummies were collected and placed separately in Petri dishes (3.5 cm in diameter). Newly emerged (within 0–12 h) *Aphidius* were transferred to larger Petri dishes (9 cm in diameter and 2 cm in height) for another 24 h, and the two groups were divided by sex. Cotton balls dipped in a 25% aqueous solution of sucrose were constantly supplied as the diet for adult wasps. Approximately 500 pairs of antennae from each sex were collected for RNA sequencing. In total, for each replication of qRT-PCR, 100 antennae, 50 heads, 50 thorax, 50 abdomens, and 300 legs were collected. Three replicates were conducted for sampling. The dissected tissues were immediately frozen in liquid nitrogen and stored at −80°C.

### Total RNA Extraction and Synthesis of the First Chain of cDNA

Total RNA was extracted using TRIzol reagent and combined with micro total RNA extraction kit (Tianmo, Beijing, China) following the manufacturer’s instructions. The frozen tissues were homogenized with a liquid nitrogen cooled mortar and ground with a pestle into very fine dust. Homogenized tissues were treated with 1 ml of TRIzol reagent (Invitrogen, Carlsbad, CA, United States). RNA degradation and contamination were monitored on 2% agarose gels. RNA purity was checked using a Nanodrop ND-1000 spectrophotometer (NanoDrop products, Wilmington, DE, United States). The RNA concentration was measured using a spectrophotometer RNA Nano 6000 Assay Kit of the Agilent Bioanalyzer 2,100 system (Agilent Technologies, CA, United States). Individual total RNA was isolated and cDNA was synthesized using the TRUEscript RT kit (LanY Science & Technology, Beijing, China) following the manufacturer’s protocol.

### Transcriptome Sequencing, Assembly and Functional Annotation

A total of 3 μg of RNA sample with standard quality ratios (1.8 < OD260/280 < 2.1) was purified using poly-T oligo-attached magnetic beads. Divalent cations under elevated temperature in NEBNext First Strand Synthesis Reaction Buffer (5×) were used for fragmentation. Single-stranded (ss) cDNA was synthesized using a random hexamer primer, M-MuLV Reverse Transcriptase and DNA Polymerase I and RNase H (NEB, United States). The 3′ ends of the DNA fragments were adenylated and the NEBNext Adaptor was ligated to the fragments for hybridization. The library fragments were purified with the AMPure XP system (Beckman Coulter, Beverly, MA, United States) to size select cDNA fragments ∼150–200 bp in length. Then, 3 μl of USER Enzyme (NEB, United States) was used with size-selected, adaptor-ligated cDNA at 37°C for 15 min followed by 5 min at 95°C prior to PCR. PCR was performed with Phusion High-Fidelity DNA polymerase, Universal PCR primers and Index (X) Primer. The products were purified (AMPure XP system), and library quality was assessed using the Agilent Bioanalyzer 2,100 system (Agilent Technologies, CA, United States). Clustering of the index-coded samples was performed on a cBot Cluster Generation System using TruSeq PE Cluster Kit v3-cBot-HS (Illumina, China) according to the manufacturer’s instructions. The library preparations were sequenced on an Illumina HiSeq 2,500 platform and paired-end reads (the sequencing strategy was PE125) were generated after cluster generation. After sequencing, the raw reads were processed to remove low quality and adaptor sequences by ng_qc, and then assembled into unigenes using Trinity r20140413p1 min_kmer_cov:2 and other default parameters (Grabherr et al., 2011). Then the unigenes were annotated using seven databases, including the nonredundant protein sequence (Nr, e-value = 1e^−5^), nonredundant nucleotide (Nt, e-value = 1e^−5^), Pfam (e-value = 0.01), Clusters of Orthologous Groups (KOG/COG, e-value = 1e^−3^), Swiss-Prot (e-value = 1e^−5^), Kyoto Encyclopedia of Genes and Genomes (KEGG, e-value = 1e^−10^) and Gene Ontology (GO, e-value = 1e^−6^) databases.

### OBP Gene Prediction and Identification

The available sequences of *OBPs* from Hymenoptera species were used as “query” sequences to identify candidate unigenes that code OBPs in the *A. gifuensis* antennal transcriptome with the TBLASTn program with an e-value threshold of 10^–5^. The sequences that fit the criteria were considered candidate OBPs. The open reading frames were searched by ORF finder (http://www.ncbi.nlm.nih.gov.orffinder/). The putative N-terminal signal peptides were predicted using the SignalP V4.1 program (http://www.cbs.dtu.dk/services/SignalP-4.1/) following the default parameters. Alignments of amino acid sequences were performed with Clustal Omega (https://www.ebi.ac.uk/Tools/msa/clustalo/) and edited using DNAMAN (Lynnon Biosoft San Ramon, CA, United States) software. According to the DEG results, the mean FPKM values for each gene in the antennae of males and females were then log-transformed [“log_2_ (FPKM +1)”]. A heat map was generated using TBtools (Chen et al., 2020). A phylogenetic tree was constructed by MEGA11 using the maximum likelihood method with a LG + mode to analyze the relationship of OBPs among species and reveal clues of their function (Tamura et al., 2021). Values indicated at the nodes are bootstrap values based on 1,000 replicates presented with 95% cutoff. The orthologous protein sequences from the genomes and transcriptomes of the following Hymenoptera species were used in the analysis: *Apis mellifera* (Forêt and Maleszka, 2006 **)**; *M. mediator* (Zhang et al., 2009; Peng et al., 2017 **)**; *M. pulchricornis* (Sheng, et al., 2017 **)** and *Aulacocentrum confusum* (Li et al., 2021). The amino acids of the sequences used are listed in Supplementary Material S1. A circular phylogenetic tree was then generated and taxonomically color-coded using the online tool iTOL (https://itol.embl.de/itol.cgi). To identify the sequences of all candidate *AgifOBPs*, gene-specific primers (Supplementary Table S3) were designed with Primer 5.0 (http://frodo.wi.mit.edu/primer5/). Polymerase chain reactions were conducted on an Eppendorf Mastercycler^®^ gradient PCR machine using 2×TransStart FastPfu PCR SuperMix (Trans, Beijing, China) and antennal cDNA as a template. An initial denaturation step at 95°C for 5 min followed by 35 cycles of 95°C for 35 s, 58°C as a melting temperature for 35 s, and 72°C for 45 s, and a final extension at 72°C for 10 min. The PCR products were electrophoresed on 2% agarose gels and stained with ethidium bromide to ensure that the correct products were amplified. All targeted PCR products were purified using the AxyPrep PCR clean up Kit (CORING, Jiangshu, China), and then cloned into the pEASY Blunt clone vector (Trans, Beijing, China). After transformation of *Escherichia coli* DH5α competent cells with the ligation products, positive colonies were selected by PCR using the plasmid primers M13 F and M13 R and sequenced at San bo Biotech (Beijing, China). Individual clones confirmed to contain the desired sequence were incubated in LB/ampicillin medium.

### Spatial Expression Pattern of AgifOBPs

To explore the expression characteristics of the *AgifOBPs*, RT-qPCR with an ABI 7500 real-time PCR system (Applied Biosystems Fosters City CA, United States) was conducted with cDNAs prepared from each tissue of male and female *Aphidius*. Briefly, 0.6 µl of both forward primer (10 μmol/L) and reverse primer (10 μmol/L) (Supplementary Table S4) were used in a 20-µl reaction containing 10 µl of 2x SuperReal PreMix Plus, 2 µl of cDNA (from 250 ng of total RNA), 0.4 µl of 50x ROX reference dye, and 6.4 µl of ribonuclease-free ddH_2_O following the instructions provided with the SuperReal PreMix Plus (SYBR Green) kit (FP205) (Tiangen, Beijing, China). The PCR program was as follows: initial 15-min step at 95°C, 40 cycles of denaturation at 95°C for 15 s, annealing at 60°C for 32 s and elongation at 72°C for 1 min and finally a 10-min step at 72°C. For melting curve analysis, a dissociation step cycle was added automatically. The amplification efficiency was calculated using the equation: E = [10^(-1/slope)-1] ×100%, in which the slope was derived by plotting the cycle threshold (Ct) value against five 2-fold serial dilutions. Only primers with 95–105% amplification efficiencies were used for subsequent data analysis. Relative quantification was performed according to the 2^−ΔΔCt^ method **(** Livak and Schmittgen, 2001). β-Actin and (nicotinamide adenine dinucleotide) NADH were used as reference genes to normalize the data. All qRT-PCR analyses were performed in three technical and biological replications.

### Prokaryotic Expression and Purification of AgifOBP7 and AgifOBP9

The prokaryotic expression and purification procedures were consistent with previous studies (Prestwich, 1993; Wang et al., 2021a). Gene-specific primers were designed to clone the full-length cDNAs encoding mature AgifOBP proteins. The PCR products were first cloned into the pEASY-T1 clone vector (TransGen Biotech, Beijing, China), and then excised and subcloned into the bacterial expression vector pET28a (+) (Novagen, Madison, WI) between the Nde I and EcoR I restriction sites, and reconstructed plasmids were verified by sequencing. The recombinant AgifOBP7 and AgifOBP9 in the present study contain no histidine-tagged peptide at the N-terminus.

Protein expression was induced by adding isopropyl-1-thio-b-D-galactopyranoside (IPTG) to a final concentration of 1 mM when the culture reached an OD_600_ value of 0.6. Cells were incubated for an additional 12 h at 28°C and then harvested by centrifugation and sonicated at a low temperature (ice-water mixture). After centrifugation, the bands obtained were checked by 15% SDS–PAGE for their correspondence to the predicted molecular masses of the proteins. They were solubilized according to protocols for the effective rebuilding of the recombinant OBPs in their active forms (Prestwich, 1993). The soluble proteins were then purified by anion-exchange chromatography with RESOURCE Q15 HP column (GE HEALTH CARE, United States) and gel filtration [Superdex 75 10/300 GL column (GE HEALTH CARE, United States)], The crude extracts were passed over a pre-equilibrated RESOURCE Q15 HP column (20 mM Tris-HCl, pH 8.5), and then washed and eluted with Buffer B (20 mM Tris-HCL, 1 M NaCl, pH 8.5). And finally with two rounds of gel filtration through a Superdex 75 10/300 GL column., those eluted proteins were collected and analyzed by 15% SDS-PAGE, and then, several successive dialyzes were performed: 1) at 4°C for 3 h, against 2 L of storage buffer (20 mM Tris-HCl, pH 8.5), 2) at 4°C for 3 h, against 2 L storage buffer (20 mM Tris-HCl, pH 8.5), and 3) at 4°C against 2 L of storage buffer (20 mM Tris-HCl, pH 8.5) overnight. Finally, the desalted protein samples were ultracentrifuged for 30 min using 3-kDa ultrafiltration at 4°C, and 5,000 rpm. Protein samples were analyzed by SDS-PAGE after every purification step, the concentration of purified protein was determined by a Protein Assay kit (Qubit™ Protein Assay kit, Q33211, Invitrogen), and the purified AgifOBPs were analyzed by mass spectrometry (LC–MS). The purity and concentration of the soluble proteins were evaluated using SDS–PAGE. Finally, stock solutions of AgifOBP7 and AgifOBP9 were collected and kept at −20°C in Tris–HCl (50 mM, pH 7.4).

### Fluorescence Competitive Binding Assays

To investigate the ligand-binding property of two AgifOBPs, five groups of competitive ligands were used: 1) aphid alarm pheromone components, including EBF, (-)-α-pinene, (-)-β-pinene and (+)-limonene which are released by other aphids following natural enemy predation or physical damage (Francis et al., 2005; Song et al., 2021b), 2) main components of the aphid sex pheromone: (4aSR,7SR,7aRS)-Nepetalactone; 3) green leaf volatiles of wheat: (Z)-3-hexen-1-ol; 4) aphid-induced plant volatiles (methyl salicylate, and 6-methyl-5-hepten-2-one); and 5) an EBF derivative artificial chemical, namely CAU-II-11, ((E)-3,7-dimethylocta-2,6-dien-1-yl-2-hydroxy-3-methoxybenzoate), which showed a high affinity for aphid EBF- binding proteins (OBP3/7/9, Qin et al., 2020), and was used to investigate the binding properties of purified AgifOBP7 and AgifOBP9. The classes, CAS numbers and purity of the chemicals used in this study are listed in Table 1.

**TABLE 1:** Binding affinities of AgifOBP7 and AgifOBP9 for candidate ligands, evaluated in displacement binding assays using the fluorescent probe, 1-NPN.

				OBP7		OBP9	
No	Code	CAS	Purity	IC50	Ki(μΜ)	IC50	Ki(μΜ)
1	(E)-β-Farnesene	18,794–84–8	≥85%	>30	20.30 ± 1.99	17.83 ± 1.69	4.60 ± 0.43
2	(-)-α-Pinene	80–56–8	≥95%	>30	>30	24.10 ± 3.42	6.22 ± 0.88
3	(-)-β-Pinene	19,902–08–0	≥99%	>30	>30	11.86 ± 1.27	3.06 ± 0.33
4	(+)-Limonene	138–86–3	≥95%	>30	>30	12.85 ± 0.25	3.32 ± 0.06
5	(4aSR 7SR 7aRS)-Nepetalactone	21,651–62–7	≥80%	>30	16.12 ± 3.49	9.03 ± 0.31	2.33 ± 0.08
6	6-Methyl-5-hepten-2-one	110–93–0	≥99%	>30	>30	22.32 ± 3.38	5.76 ± 0.87
7	cis-3-Hexenol	928–96–1	≥97%	>30	>30	17.01 ± 0.33	4.39 ± 0.09
8	Methyl salicylate	119–36–8	≥99%	>30	>30	13.31 ± 2.99	3.43 ± 0.77
9	CAU-II-11	-	≥98%	18.56 ± 1.73	8.50 ± 0.73	4.15 ± 0.33	1.07 ± 0.08

Fluorescence intensity was recorded in a right angle configuration on a Lengguang 970CRT spectrofluorimeter (Shanghai Jingmi, China) at room temperature using a 1 cm light path fluorimeter quartz cuvette. A slit width of 10 nm was selected for both excitation and emission. The measured fluorescence intensities were corrected for both blank signals due to protein emission and scattered excitation light. The spectral data were processed using the software 970CRT 2.0l. Fluorescence binding experiments were conducted in 50 μM Tris-HCl buffer, pH 7.4, at room temperature. The binding affinity for N-phenyl-1-naphthylamine (1-NPN) was determined by adding aliquots of a 1 mM stock solution of 1-NPN dissolved in spectrophotometric grade methanol into a 2 μM protein sample. The fluorescence of 1-NPN was excited at 337 nm, and emission was recorded between 350 and 500 nm. Spectra were recorded with a high-speed scan. All ligands used in competitive experiments were dissolved in spectrophotometric grade methanol. In competition assays, aliquots of the competing ligands were added into a 2 μM protein solution in the presence of a given concentration of 1-NPN. To estimate the binding affinities of each AgifOBP for a variety of different ligands, we monitored the decrease in 1-NPN fluorescence due to the ability of different odorants to displace 1-NPN and determined the Ki value for each compound. To determine the dissociation constants, the intensity values corresponding to the maximum fluorescence emissions were plotted against the cumulative 1-NPN concentration. The amount of bound ligand was calculated from the fluorescence intensity values by assuming that the protein was 100% active, with a stoichiometry of 1:1 protein: ligand at saturation. The curves were linearized using Scatchard plots. The value of K_1-NPN_ was estimated on a direct plot by nonlinear regression with an equation corresponding to a single binding site using Prism 7 (GraphPad Software, Inc., United States), and the IC_50_ was defined as the concentration of a competitor that caused a 50% reduction in fluorescence intensity. The dissociation constants of the inhibitors (Ki) were calculated according to the formula Ki = [IC50]/(1+[1-NPN]/K_1-NPN_), in which [1-NPN] represents the free 1-NPN concentration and K_1-NPN_ represents the dissociation constant for AgifOBPs/1-NPN (Ban et al., 2003; Zhong et al., 2012; Sun et al., 2016; Fan et al., 2017; Qin et al., 2020; Wang et al., 2021b). All fluorescence competitive binding assays were performed in three independent replicates, and Ki dates are present as means ± SD.

### Statistical Analyses

For qRT-PCR analyses, the differences between means of biological replicates were tested using two-way ANOVA followed by multiple comparisons tests regardless of rows and columns using GraphPad Prism version 7.0.0 for Windows (GraphPad, Software, San Diego, California United States, www.graphpad.com). Differences between means for experiments with more than two treatments were distinguished using Tukey’s honestly significant difference (HSD) test at the *p* < 0.05 significance level.

## Results

### Overview of Transcriptomes

A total of 2.22 and 2.30 million raw reads were obtained from *A. gifuensis* antennae libraries from females and males, respectively. The data presented in the study that was deposited in the Sequence Read Archive (SRA) repository, the accession number are SRR18251541 and SRR18251542. After removal of low-quality, adaptor, and contaminating sequences, 3.31 and 3.03 million clean reads were retained (Supplementary Table S1) and assembled into 81,235 distinct transcripts (mean length = 661 bp) and 65,854 unigenes (mean length = 568 bp). The length distribution presented in (Supplementary Figure S1). In total, 18,408 (27.95% of all 65,854 unigenes), 5,625 (8.54%), 7,551 (40.92%), 12,484 (18.95%), 15,070 (22.88%), 15,951 (24.22%) and 9,462 (14.36%) transcripts from *A. gifuensis* antennae were annotated using the Nr, Nt, KO, Swiss-Prot, Pfam, GO and KOG databases, respectively (Supplementary Table S2). The most abundant GO terms were biological process terms, with *AgifOBP3* corresponding to the cellular process and *AgifOBP15* grouped with the membrane. The cluster for cellular process was the second largest group. Most transcripts that corresponded to molecular function were related to binding and catalytic activity (Supplementary Figure S2). In the KOG classification, unigenes clustered into 26 categories (Supplementary Figure S3). Among these categories, general function prediction was the dominant category, followed by signal transduction and posttranslational modification, protein turnover and chaperon. All the unigenes annotated in the KO database were assigned to the 5 biological pathways described in the KEGG database: cellular processes, environmental information processing, genetic information processing, metabolism, and organismal systems (Supplementary Figure S4). The most common pathway was metabolism followed by genetic information processing, organismal systems and cellular processes. Signal transduction was involved in 940 genes in the environmental information processing group.

### OBP Prediction and Phylogenetic Analysis

**FIGURE 1:**
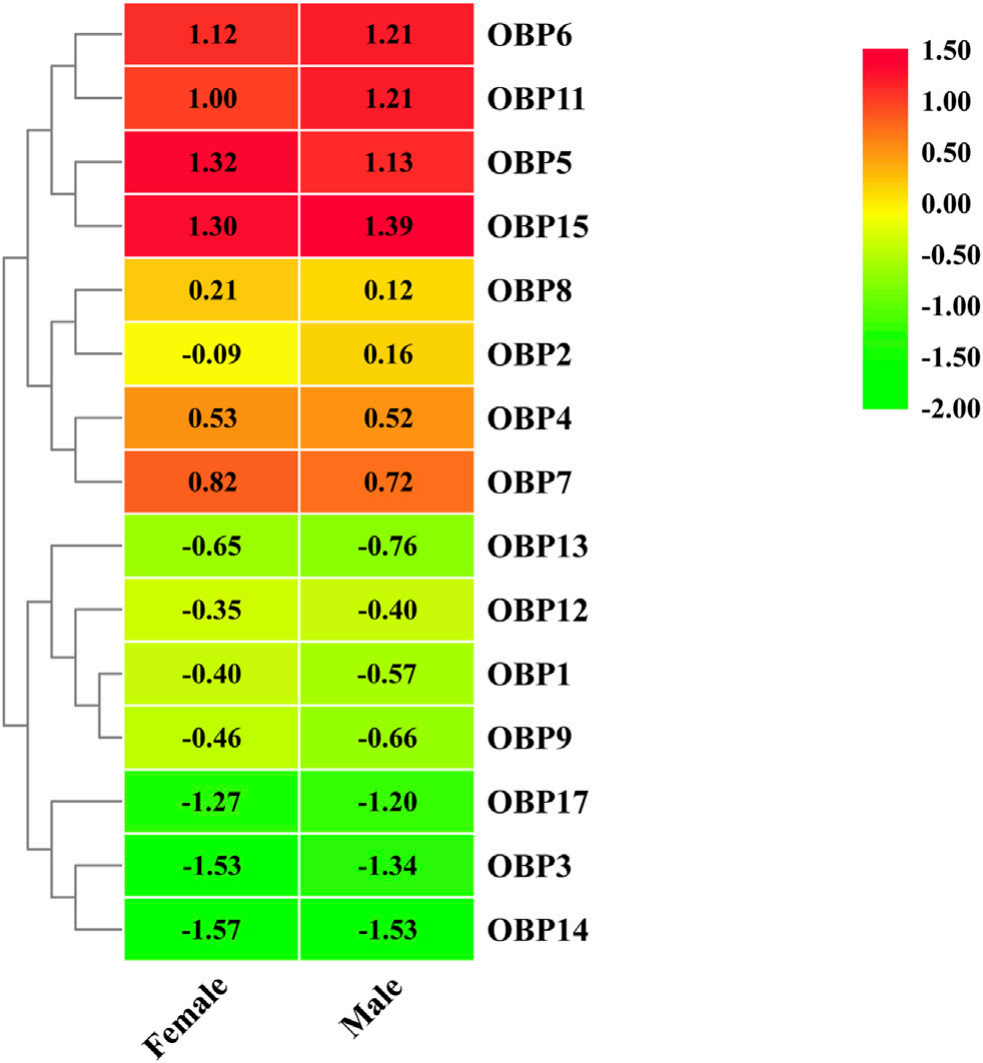
Heatmap of differentially expressed OBP genes between females and males based on FPKM values of antennae transcriptomes.

**FIGURE 2:**
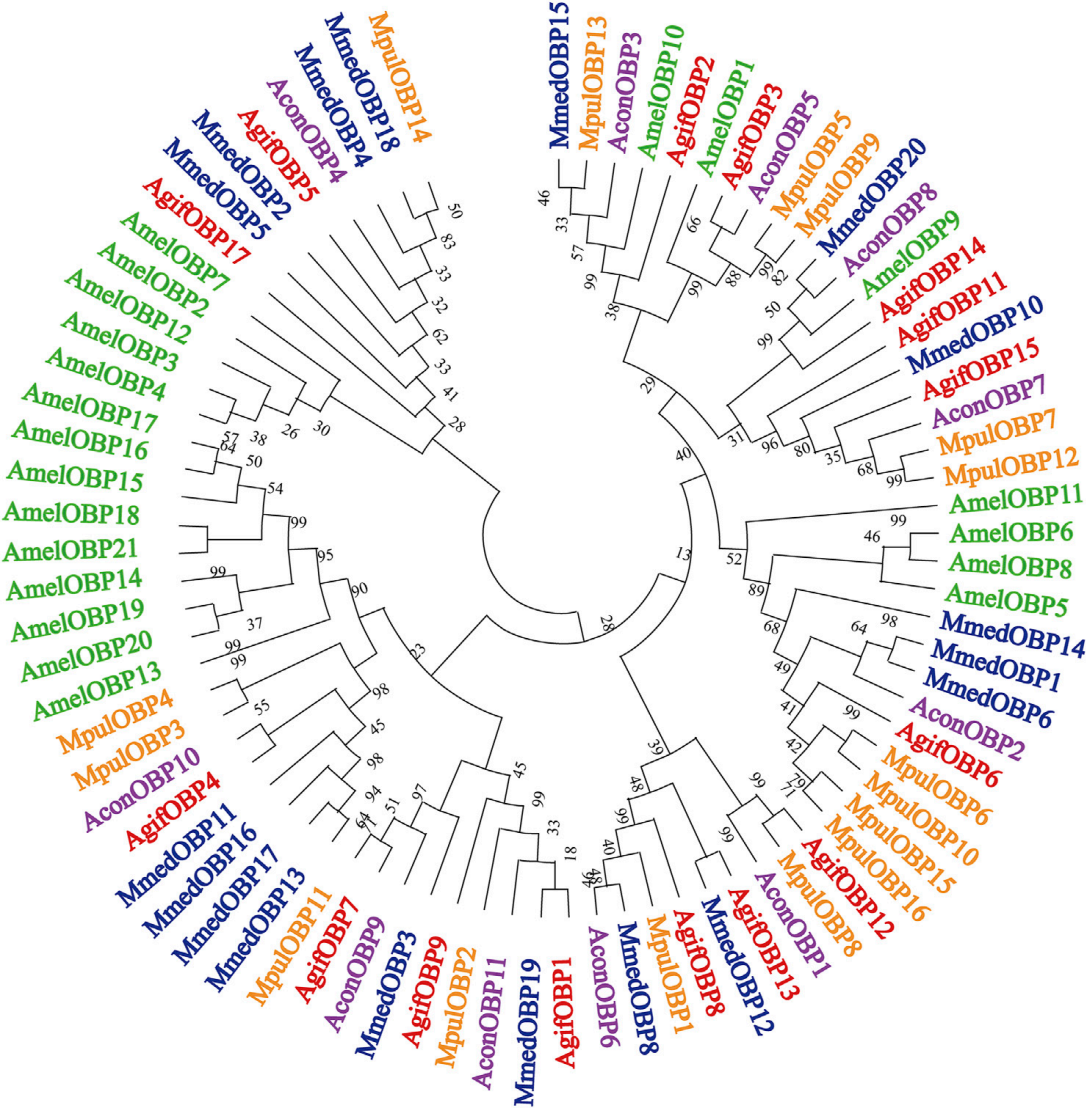
Phylogenetic relationships of target parasitoid putative OBPs and 66 putative other hymenopteran OBPs; detailed relationships of the putative *AgifOBPs* (in red), *MmedOBPs* (in blue), *AmelOBPs* (in green), *MpulOBPs* (in orange), and *AconOBPs* (in purple). The trees were constructed with MEGA 11 using an LG + model and bootstrap support was calculated with 1,000 rapid bootstrap replicates with a 95% cutoff. The species names are abbreviated with four letters, and their full names with all accession numbers of the OBP amino acid sequences are provided in Supplementary Material S1.

Fifteen putative OBPs with complete open reading frames were predicted from the antennal transcriptome data. We mainly named them following Fan’s work (Fan et al., 2018)*. AgifOBP10* with a partial ORF reported by Fan is missing here. All OBP transcripts were confirmed by molecular cloning, followed by sequencing, except for *AgifOBP14.* All 15 OBPs have the characteristic of insect OBP sequence motif (Yuan et al., 2015), and 12 *AgifO*BPs (*AgifOBP1-3,5–9,11,13,14,15*) of them have the classic OBP Cys motif (C_1_-X_22-32_-C_2_-X_3_-C_3_-X_36_-_46_-C_4_-X_8_-_14_-C_5_-X_8_-C_6_) (Xu et al., 2009), while 3 *AgifOB*Ps (*AgifOBP4/12/17*) belong to the minus-C OBP Cys motif with four or five conserved cysteines (Supplementary Figure S5). The heatmap in Figure 1 illustrates that OBP5, OBP6, OBP11, and OBP15 were highly expressed genes in both sex antennae but OBP3/14/17 showed relatively low expression levels. The phylogenetic tree of Hymenoptera OBPs was built using MEGA11 (maximum likelihood method with an LG model) OBP sequences from 5 different species (*A. gifuensis*, *A. mellifera*, *M. mediator*, *M. pulchricornis* and *A. confusum*). *A. gifuensis* OBPs are clustered together to form three homologous subgroups (lineages). Among them, OBP1, OBP5, OBP7, OBP9 andOBP17 were in one subgroup, OBP2, OBP3, OBP6, OBP8 and OBP11-OBP15 were in the other subgroup, and OBP4 fell into the third subgroup. The results showed that AgifOBP*s* almost spread across in clades without species specificity (Figure 2). Among these AgifOBPs, AgifOBP4 was found in the MpulOBP4 clade. AgifOBP6 exhibited a rather high similarity to other orthologs such as AmelOBP6, MmedOBP6 and MpulOBP6. AgifOBP8 also showed a high similarity to MmedOBP8 and MpulOBP8 (Figure 2).

### Spatial Expression Pattern of *AgifOBPs*

**FIGURE 3:**
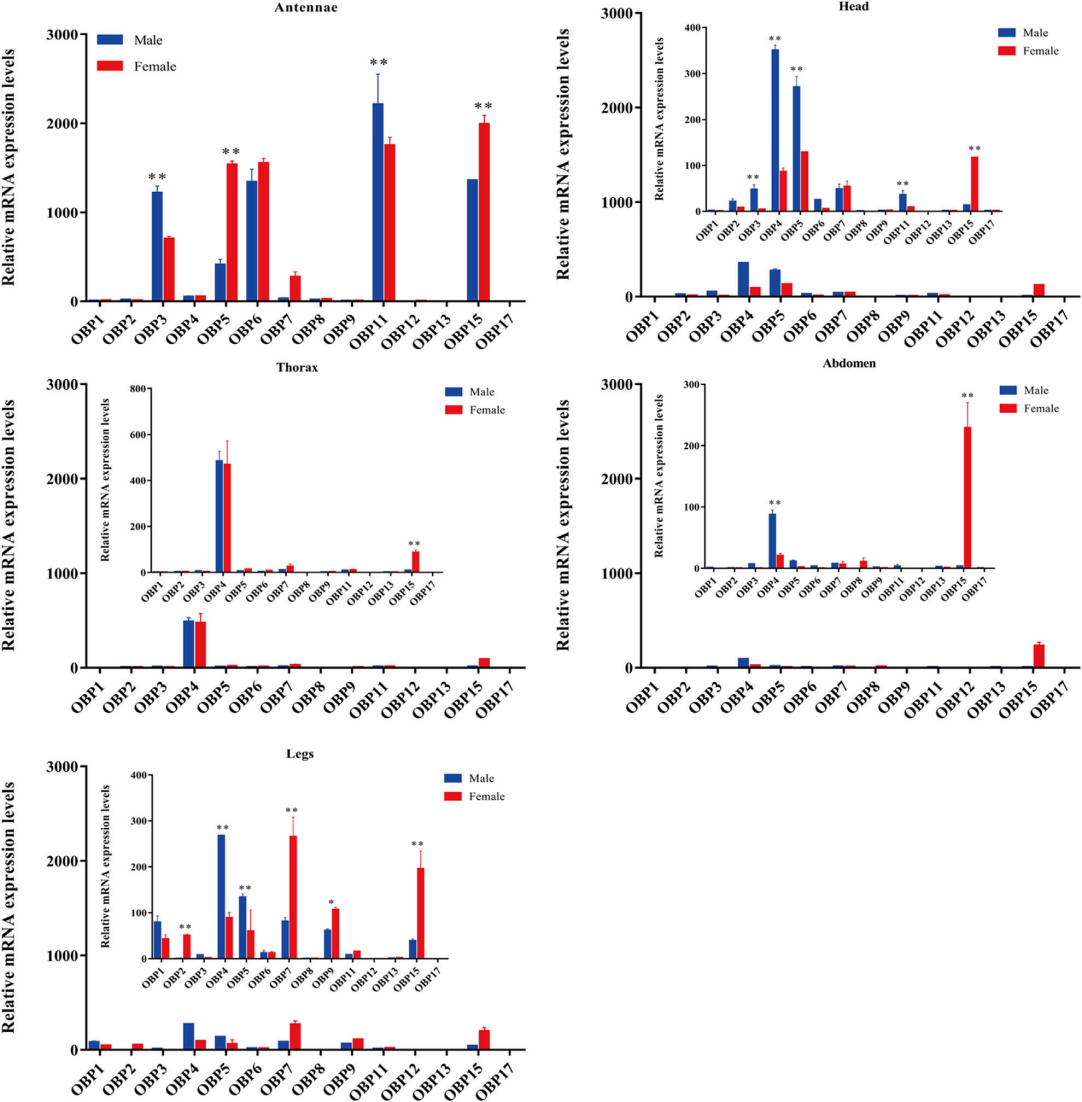
The relative expression patterns of different *AgifOBP* genes in different tissues of males and females as measured by quantitative real-time polymerase chain reaction. The fold changes are relative to the transcript levels of OBP13 in the male thorax. The NADH and ACTIN genes were used as references to normalize the expression of each tested gene. The data are presented as the mean ± SD. The asterisk * and ** above the bars indicate significant differences at *p* < 0.05; and *p* < 0.01, respectively, according to two-way ANOVA.

**FIGURE 4:**
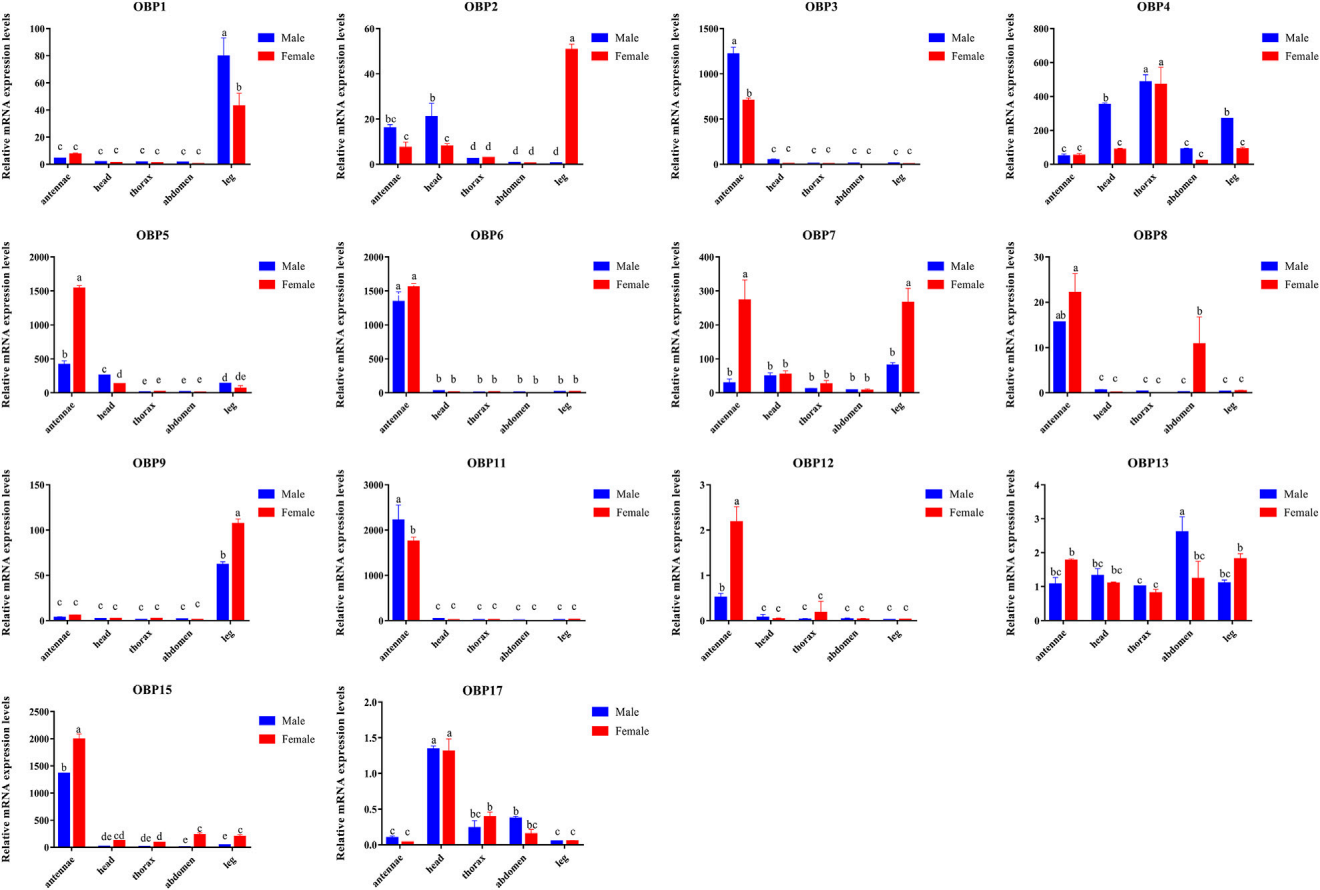
Relative expression of *AgifOBP* genes in the different tissues of male and female *A. gifuensis* as measured by quantitative real-time polymerase chain reaction. Relative fold changes were normalized to the transcript levels in the male thorax. The NADH and ACTIN genes were used as references to normalize the expression of each tested gene. The standard error is represented by the error bar (*n* = 3) and the different lowercase letters (a, b, c, d, e) indicate significant differences in transcript abundances (two-way ANOVA followed by Tukey’s HSD multiple comparison test, *p* < 0.05).

Compared to other tissues or organs, 8 of the 14 OBPs, namely, *AgifOBP3, AgifOBP5, AgifOBP6, AgifOBP7, AgifOBP8, AgifOBP11, AgifOBP12* and *AgifOBP15,* maintained higher expression in antennae (Figures 3, 4; *p* < 0.05). *AgifOBP17* was highly expressed in the head. *AgifOBP1/2/7/9* were expressed in legs with significantly higher expression levels (Figures 3, 4, *p* < 0.05). The other two *OBPs*, *AgifOBP4* and *AgifOBP13* were widely expressed among tissues and organs.

Specifically, *AgifOBP3/5/6/11/12/15* were specifically expressed in antennae. Among them, the expression levels of AgifOBP3/11 were even higher in male antennae. However, *AgifOBP12/15* were even higher in female antennae, and *AgifOBP6* showed no difference in antennae of both sexes. Moreover, *AgifOBP1/2/4/5/7/9/15* showed relatively higher expression levels in legs (Figures 3, 4). Among them, *AgifOBP2* was specifically expressed in female legs. And *AgifOBP7/9/15* were expressed at comparatively higher levels in female legs. In contrast, *AgifOBP1/4/5* were expressed at higher levels in male legs. Notably *AgifOBP7* was female specific and was expressed directly in female antennae and legs. In males, *AigfOBP8* was specifically expressed in antennae. In females, it was relatively highly expressed in both the antennae and abdomen. We also found that the highest level of *AgifOBP17* was in the heads of both sexes. Although both *AgifOBP4* and *AgifOBP13* were widely expressed, *AgifOBP4* showed an even higher expression level in thoraxes of both females and males. *AgifOBP13* expression was significantly higher in the male abdomen. In addition, *AgifOBP1* and *AgifOBP9* were specifically or highly expressed in the legs of both male and female *A. gifuensis. AgifOBP2* was significantly expressed in the legs of females (Figure 4, *p* < 0.05).

In summary, *AgifOBP3/5/6/11/12/15* were antennal specifically expressed OBPs. *AgifOBP2/9* were specifically expressed OBPs in legs. *AgifOBP17* is an OBP specifically in the head (Figure 3, *p* < 0.05). In addition, *AgifOBP5/7* were female specific OBPs. *AgifOBP8* expression was significantly higher in the antennae of males and in both the antennae and abdomen of females (Figure 4, *p* < 0.05).

### Expression and Purification of AgifOBP7 and AgifOBP9

**FIGURE 5:**
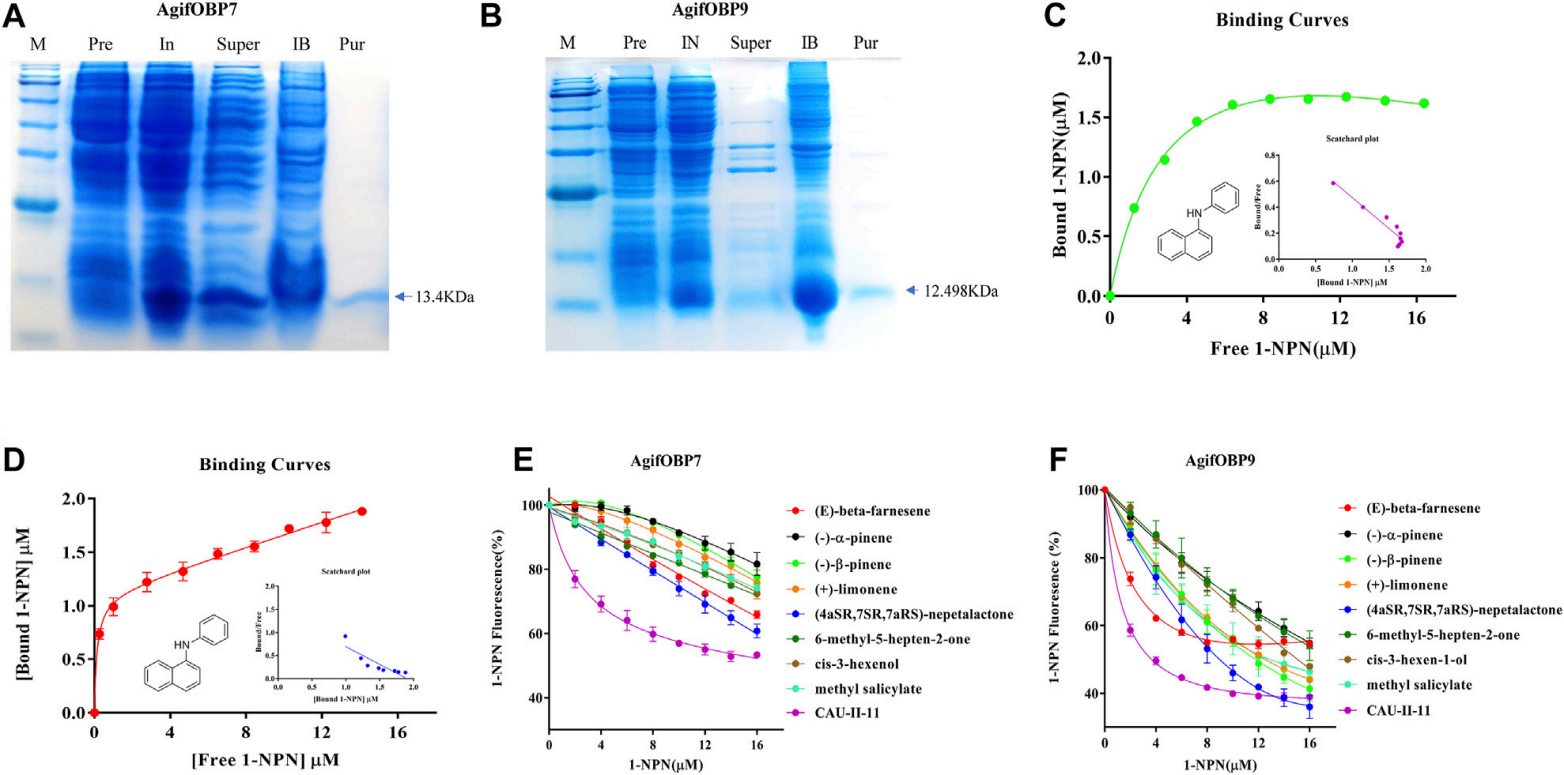
Expression and binding properties of AgifOBP7 and AgifOBP9 with candidate ligands. **(A)** Expression and purification of AgifOBP7, **(B)** Expression and purification of AgifOBP9. Line M: molecular weight PR 1910 (11–180 KDa) Marker, 11, 17, 25, 35, 48, 63, 75, 100, 135, 180 KDa; IN: Induced pET-28a (+)/AgifOBP7/9; Super: pET-28a (+)/AgifOBP7/9 Supernatant; IB: pET-28a (+)/AgifOBP7/9 Inclusion body; Pur: Purified pET-28a (+)/AgifOBP7/9 without His-tag. **(C,D)** Binding curves of AgifOBP7 and AgifOBP9 with N-phenyl-1-naphthylamine (1-NPN) in 50 mM Tris-HCl buffer (pH 7.4). **(E,F)** Competitive binding curves of AgifOBP7 and AgifOBP9 to components of aphid alarm pheromones ((E)-beta-farnesene, (-)-α-pinene, (-)-β-pinene, (+)-limonene, (E)-beta-farnesene derivative (CAU-II-11); aphid sexual pheromone (4aSR 7SR 7aRS)-nepetalactone, the volatiles of wheat green leaf (cis-3-hexen-1-ol) and aphid induced plant main volatiles (methyl salicylate, 6-methyl-5-hepten-2-one). A mixture of the recombinant protein and N-phenyl-1-naphthylamine (1-NPN) in 50 mM Tris-HCl buffer (pH 7.4) at the concentration of 2 μM was titrated with 1 mM solutions of each competing ligand to a final concentration range of 2–16 μM. Fluorescence values are presented as percent of the values in the absence of competitor. Date are the means ± SD of three independent experiments.

AgifOBP7 and AgifOBP9 were successfully expressed in the inclusion bodies using a bacterial system. After a dissolving and refolding treatment, the refolded AgifOBP7 and AgifOBP9 were purified with yields of 0.25 mg/ml as soluble proteins (Figures 5A,B). More than 15 mg of purified AgifOBP7 and AgifOBP9 was obtained using RESOURCE Q15 affinity columns, with the His-tag removed. The theoretical molecular weight values for AgifOBP7 and AgifOBP9 were very close to the measured values (AgifOBP7, 13.401 kDa; AgifOBP9, 12.498 kDa). The purified protein samples were further identified by LC–MS/MS (data not shown).

### Fluorescence Competitive Binding Assays

To investigate the role of two OBPs in the odor perception of aphids, we chose alarm pheromones (EBF, (-)-α-pinene, (-)-β-pinene, (+)-limonene, EBF derivative (CAU-II-11), aphid sexual pheromones (4aSR 7SR 7aRS)-nepetalactone as well as volatiles of wheat green leaf (cis-3-hexen-1-ol) and aphid induced plant main volatiles (methyl salicylate, 6-methyl-5-hepten-2-one) as the candidate ligands for fluorescence competitive binding assays (Table 1). We first tested the binding affinities of both OBPs to the fluorescent probe N-phenyl-1-naphthylamine (1-NPN) as previously reported (Qiao et al., 2009; Fan et al., 2017). The dissociation constants of AgifOBP7/1-NPN and the AgifOBP9/1-NPN complex were 1.69 ± 0.27 µM and 0.69 ± 0.24 µM respectively (Figures 5C,D).

In a subsequent experiment, we used a fluorescence competitive binding assay to determine the binding affinities of AgifOBP7 and AgifOBP9 to different odorants. Based on the binding curves, we calculated the median inhibitory concentration (IC50) and dissociation constant (Ki) values (Table 1). Among the tested odorants, EBF, (-)-α-pinene, (-)-β-pinene, (+)-limonene, (4aSR 7SR 7aRS)-nepetalactone; cis-3-hexen-1-ol, methyl salicylate and 6-methyl-5-hepten-2-one displayed relatively high binding affinities (Ki < 10 µM) to AgifOBP9 (Figure 5F). Interestingly, among all the tested odorants, CAU-II-11 bound most strongly (Ki = 1.07 ± 0.08 µM) to AgifOBP9 (Table 1), which is the derivative of EBF (Ki = 4.60 ± 0.43 µM). However, this was not the case with AgifOBP7, which only displayed weak binding with EBF (Ki = 20.30 ± 1.99) and (4aSR 7SR 7aRS)-nepetalactone (Ki = 16.12 ± 3.49), not much with (-)-α-pinene, (-)-β-pinene, (+)-limonene, cis-3-hexen-1-ol, methyl salicylate and 6-methyl-5-hepten-2-one. CAU-II-11, like AgifOBP9, showed the strongest binding affinity (Ki = 1.07 ± 0.08 µM) to AgifOBP7 among the examined odorants (Figure 5E; Table 1).

## Discussion

Odorant-binding proteins are classically defined as olfactory soluble proteins (Vogt, R. G., & Riddiford, L. M, 1981; Pelosi, 2006) and play an essential role in habitat searching and finding suitable mates. With the increase in insect genome projects and transcriptome sequencing projects, large numbers of OBPs have recently been identified in different insect species. In the present study, we constructed a cDNA library from the antennae of the endoparasitoid *A. gifuensis* for transcriptome sequencing and categorized the potential function of the odorant binding protein genes by bioinformatics approaches.

### OBP Prediction, Cloning and Phylogenetic Analysis

Fourteen OBPs in the *A. gifuensis* antennae transcriptome were identified in the present study. This number is similar to those in *A. mellifera* (Forêt and Maleszka, 2006), *M. mediator* (Zhang et al., 2009; Peng et al., 2017), *Meteorus pulchricornis* (Sheng et al., 2017), *Cotesia vestalis* (Zhou et al., 2021) and *Aulacocentrum confusum* (Li et al., 2021), therefore indicating that there are similar OBP numbers in Hymenoptera insects.

The phylogenetic tree of these *AgifOBPs*, together with OBPs from 4 hymenopteran species, showed that the *AgifOBPs* segregate into the orthologous clades of the other species, rather than into *A. gifuensis* paralogous clades. *AgifOBP4* was found in the *MpulOBP4* clade, whereas *AconOBP4*, *MmedOBP4*, and *AmelOBP4* were clustered in the other one clade. *AgifOBP6* and *AgifOBP8* were present in the three wasps of *A. gifuensis*, *M. mediator* and *M. pulchricornis,* but their orthologs were rarely found in *Apis mellifera* (Figure 2). This also suggests that these *AgifOBPs* might play different roles in odor recognition or have roles other than olfaction. The comparatively conserved OBPs in hymenoptera wasps, particularly in parasitoid wasps implied that their function could be limited to the common olfactory physiology of these insects. Some study results on natural enemies of aphids support this hypothesis. For example, aphid OBP7 orthologs have been widely reported to have their affinities with the alarm pheromone EBF (Sun et al., 2012a; Zhong et al., 2012; Fan et al., 2017; Qin et al., 2020). *CpalOBP10* in lacewing *Chrysopa pallens*, an aphid predator, belongs to the same lineage as aphid OBP7 such as in *S. avenae* and in *A. pisum,* and its affinity for EBF was also consistent with that of aphid OBP7 orthologs (Li et al., 2017; Li et al., 2019).

### Spatial Expression Pattern

The spatial expression profile of *AgifOBPs* was verified using qPCR. Our data revealed that five *OBPs*, namely *AgifOBP3*, *AgifOBP5*, *AgifOBP6*, *AgifOBP11*, and AgifOBP15, were expressed at a high level in the antennae (Figures 3, 4), while four *OBPs*, *AgifOBP2*, *AgifOBP4*, *AgifOBP7* and *AgifOBP8*, were expressed at a medium level, and seven *OBPs*, *AgifOBP1*, *AgifOBP9*, *AgifOBP12/13*, and *AgifOBP17*, were expressed at a low level in the antennae (Figures 3, 4). The antennal specific *OBPs* (Figure 4) suggest their function of recognizing and binding odorants from the environment. Six *OBPs*, *AgifOBP2, AgifOBP4, AgifOBP5, AgifOBP7, AgifOBP13* and *AgifOBP17,* showed expression patterns among sensory and nonsensory organs, indicating their possible multiple functions in olfactory perception as well as other physiological processes such as development and reproduction. Both *AgifOBP1* and *AgifOBP9* showed higher expression levels in the legs than the other four tissues (antennae, heads, thorax and abdomen), which could be related to the adaptation of *A. gifuensis* during migration as we have discussed in our previous study (Xue et al., 2016), and might be involved in the procedure of taste or volatile perception or be related to olfactory sensilla on the legs (Yasukawa et al., 2010; Harada et al., 2012). A similar condition was also found for *AgifOBP5*, which is expressed in small amounts in the head and leg, in addition to being expressed abundantly in antennae.

Apart from antennae, alternatively, these OBPs expressed in other tissues may be responsible for corresponding functions. For example, *NlugOBP3* is highly expressed in the abdomen of *Nilaparvata lugens* and may be involved in juvenile hormone transport and play an important role in metamorphosis (He et al., 2011). Insect OBPs have been reported to act as carrier proteins in the male reproductive apparatus of mosquitoes (Li et al., 2008). After mating, the OBPs expressed by male moths are found on the surface of fertilized eggs, which helps the larvae to avoid cannibalistic behaviors (Sun et al., 2012b). For parasitic wasps, *AconOBP8* was reported to be expressed predominantly in the abdomen (Li et al., 2021). Similar expression patterns of OBPs in the nonolfactory tissues were observed in *Sclerodermus sp*. (Zhou et al., 2015) and *M. pulchricornis* (Sheng et al., 2017). In our present study, qPCR analysis revealed that *AgifOBP8* was also expressed in the female abdomen, and it can be speculated that *OBP8* may potentially function as a pheromone-binding protein for identifying a particular signal such as the sex pheromone component in mating or oviposition behaviors, although the active component of sex pheromone in this species is still unclear.

The results obtained by qPCR are consistent with antennal transcriptome based differential expression analysis (heatmap, Figure 1). Nonetheless, any discrepancy between qPCR and differential expression analysis results illustrate the poor performance of showing local details by omics big data analysis.

### Ligand-Binding Properties

Table 1 indicates that the proteins AgifOBP7 and AgifOBP9 have broad binding activities across the aphid alarm pheromone components, aphid sex pheromone, green leaf volatiles, aphid-induced plant volatiles and EBF derivatives. AgifOBP9 showed higher binding activities than AgifOBP7 with all the five types of compounds. Similar results have been found in its prey aphid *A. pisum*, in which ApisOBP9 also exhibited higher affinities with all the compounds than ApisOBP7 (Qin et al., 2020), although there is no evolutionary homology between the two species. For AgifOBP7 and AgifOBP9, EBF derivatives had higher binding properties than the lead EBF and other compounds. These results are consistent with studies on the characterized OBPs of ApisOBP1, ApisOBP3, and ApisOBP6-OBP10 in *A. pisum* (Sun et al., 2012a; Qin et al., 2020). Both proteins show preferential binding to several related compounds. AgifOBP7 bound the above five types of compounds from strong to weak: EBF derivative, aphid sex pheromone main component, aphid alarm pheromone component EBF, and other alarm pheromone components, green leaf volatile and induced plant volatiles. AgifOBP9, bound the above five types of compounds from strong to weak: EBF derivative, aphid sex pheromone main component, aphid alarm pheromone component (-)-β-pinene and (+)-limonene, induced plant volatile methyl salicylate, green leaf volatile (Z)-3-hexen-1-ol, alarm pheromone component EBF, induced plant volatile 6-methyl-5-hepten-2-one and alarm pheromone component (-)-α-pinene. Our results suggest that there are substantial differences in their interactions such that AgifOBP7binds strongly to aphid pheromone components and derivatives and binds weakly to the others, which is similar to OBP7 in *S. avenea* (Zhong et al., 2012); and AgifOBP9 broadly binds to all kinds of compounds, which is likely OBP9 in *M. persicae* (Wang et al., 2021a).

As the natural enemy of aphids, *A. gifuensis* locates aphids using the cues of aphid pheromones and plant volatiles (Powell et al., 1998; Kang et al., 2017). Our results indicate that AgifOBP7 is specific to aphid pheromone components and EBF derivatives, and that AgifOBP9 has a broad spectrum of binding to compounds. Other OBPs in aphid natural enemies also bound to aphid pheromone components and plant volatiles. For example, OBP3, OBP4, OBP6, OBP7, OBP9, and OBP10 in *Chrysopa pallens* bind plant volatiles and aphid alarm pheromone EBF, and, OBP10 specifically binds EBF (Li, et al., 2017; Li et al., 2019). The new OBPs from the aphid natural enemy *Eupeodes corollae*, OBP12, OBP15 and OBP16 also bound with EBF and plant volatiles, among which OBP12 and OBP15 strongly bound EBF (Wang et al., 2022).

Many natural enemies such as *Aphidius ervi*, *Aphidius uzbekistanicus*, and *Adalia bipunctata* are attracted to EBF **(** Buitenhuis et al., 2004 **)**. To confirm the functions suggested by the phylogenetic tree and tissue expression profiles, AgifOBP7 and AgifOBP9 were selected to perform a potential functional study. Overall, the odorants exhibited relatively high binding affinities (Ki < 10 µM) to *AgifOBP9* (Figure 5F; Table 1). Interestingly, among all the tested odorants, CAU-II-11 had the strongest binding affinity (Ki = 1.07 ± 0.08 µM) to AgifOBP9 (Table 1), which is the derivative of EBF (Ki = 4.60 ± 0.43 µM). This finding is in line with prior research on ApisOBP3/7/9 using CAU-II-11 (Qin et al., 2020). This result further supports that both aphid-induced volatiles as well as EBF are used by *A. gifuensis* in aphid location and that AgifOBP9 may be involved in this process.

In summary, we first predicted 15 *OBPs* based on the antennal transcriptome of both male and female *A. gifuensis*. Fourteen of these OBPs were verified by gene cloning. Furthermore, their detailed spatial expression pattern showed that most *OBPs* are mainly expressed in the sensory organs, but some are widely expressed in various tissues or organs such as the thorax and abdomen. Finally, at least one female particularly expressing OBP (AgifOBP9) showed affinity to EBF in a fluorescence competition experiment, which further indicated the likely molecular basement of sensing the aphid alarm pheromone at the molecular level in *A. gifuensis*. In addition, what cannot be ignored is the presence of *OBPs* expressed in other nonsensory organs such as the abdomen, which supports the existence of carrier transport functions other than for foreign chemicals and therefore broader ligand ranges of wasp *OBPs*. Our findings may shed insight into parasitic wasps’ olfactory sensitivity to host hints, as olfactory organs recognize pheromones and odorant substances that influence both host hunting and oviposition activities and will help us better understand parasitic wasp host forging and mating behaviors, which will aid in the strengthening and better utilization of *A. gifuensis* as a powerful and natural biocontrol strategy. As a result, we anticipate that additional research into the aforementioned topics will improve the efficacy of parasitoid-based biological control approaches against aphid pests.

## Data Availability

The data presented in the study was deposited in the Sequence Read Archive (SRA) repository, the accession number are SRR18251541 and SRR18251542.

## References

[B1] Ban L. Scaloni A. D'Ambrosio C. Zhang L. Yan Y. Pelosi P. (2003). Biochemical Characterization and Bacterial Expression of an Odorant-Binding Protein from *Locusta migratoria* . Cell Mol. Life Sci. (Cmls) 60 (2), 390–400. 10.1007/s000180300032 12678502 PMC11138600

[B2] Buitenhuis R. Mcneil J. N. Boivin G. Brodeur J. (2004). The Role of honeydew in Host Searching of Aphid Hyperparasitoids. J. Chem. Ecol. 30 (2), 273–285. 10.1023/b:joec.0000017977.39957.97 15112724

[B3] Calvello M. Guerra N. Brandazza A. D'Ambrosio C. Scaloni A. Dani F. R. (2003). Soluble Proteins of Chemical Communication in the Social Wasp *Polistes dominulus* . Cell Mol. Life Sci. (Cmls) 60, 1933–1943. 10.1007/s00018-003-3186-5 14523553 PMC11138633

[B4] Chen C. Chen H. Zhang Y. Thomas H. R. Frank M. H. He Y. (2020). TBtools: An Integrative Toolkit Developed for Interactive Analyses of Big Biological Data. Mol. Plant 13 (8), 1194–1202. 10.1016/j.molp.2020.06.009 32585190

[B5] De Moraes C. M. Lewis W. J. Paré P. W. Alborn H. T. Tumlinson J. H. (1998). Herbivore-infested Plants Selectively Attract Parasitoids. Nature 393 (6685), 570–573. 10.1038/31219

[B6] Dong W. X. Zhang F. Fang Y. L. Zhang Z. N. (2008). Electroantennogram Responses of Aphid Parasitoid *Aphidius Gifuensis* to Aphid Pheromones and Host-Plant Volatiles. Chin. J. Ecol. 27 (4), 591–595. 10.3724/SP.J.1141.2008.00438

[B7] Fan J. Xue W. Duan H. Jiang X. Zhang Y. Yu W. (2017). Identification of an Intraspecific Alarm Pheromone and Two Conserved Odorant-Binding Proteins Associated with (E)-β-farnesene Perception in Aphid Rhopalosiphum Padi. J. Insect Physiol. 101, 151–160. 10.1016/j.jinsphys.2017.07.014 28778653

[B8] Fan J. Zhang Q. Xu Q. Xue W. Han Z. Sun J. (2018). Differential Expression Analysis of Olfactory Genes Based on a Combination of Sequencing Platforms and Behavioral Investigations in *Aphidius Gifuensis* . Front. Physiol. 9, 1679. 10.3389/fphys.2018.01679 30542294 PMC6277867

[B9] Forêt S. Maleszka R. (2006). Function and Evolution of a Gene Family Encoding Odorant Binding-like Proteins in a Social Insect, the Honey Bee (*Apis mellifera*). Genome Res. 16 (11), 1404–1413. 10.1101/gr.5075706 17065610 PMC1626642

[B10] Francis F. Vandermoten S. Verheggen F. Lognay G. Haubruge E. (2005). Is the (E)-β-farnesene Only Volatile Terpenoid in Aphids? J. Appl. Entomol. 129 (1), 6–11. 10.1111/j.1439-0418.2005.00925.x

[B11] Gao X.-K. Zhang S. Luo J.-Y. Wang C.-Y. Lü L.-M. Zhang L.-J. (2018). Molecular Characterization and Ligand-Binding Properties of Six Odorant-Binding Proteins (OBPs) from Aphis Gossypii. J. Asia-Pacific Entomol. 21, 914–925. 10.1016/j.aspen.2018.07.004

[B12] Grabherr M. G. Haas B. J. Yassour M. Levin J. Z. Thompson D. A. Amit I. (2011). Full-length Transcriptome Assembly from Rna-Seq Data without a Reference Genome. Nat. Biotechnol. 29 (7), 644–652. 10.1038/nbt.1883 21572440 PMC3571712

[B13] Harada E. Nakagawa J. Asano T. Taoka M. Sorimachi H. Ito Y. (2012). Functional evolution of duplicated odorant-binding protein genes, Obp57d and Obp57e, in Drosophila. PLoS One 7 (1), e29710. 10.1371/journal.pone.0029710 22238638 PMC3253112

[B14] He P. Zhang J. Liu N.-Y. Zhang Y.-N. Yang K. Dong S.-L. (2011). Distinct Expression Profiles and Different Functions of Odorant Binding Proteins in *Nilaparvata lugens* Stål. PLoS One 6 (12), e28921. 10.1371/journal.pone.0028921 22174925 PMC3235172

[B15] Iovinella I. Dani F. R. Niccolini A. Sagona S. Michelucci E. Gazzano A. (2011). Differential Expression of Odorant-Binding Proteins in the Mandibular Glands of the Honey Bee According to Caste and Age. J. Proteome Res. 10, 3439–3449. 10.1021/pr2000754 21707107

[B16] Jiang X. Zhang Q. Qin Y. Yin H. Zhang S. Li Q. (2019). A Chromosome-Level Draft Genome of the Grain Aphid Sitobion Miscanthi. Sitobion miscanthiGigascience 8 (8), giz101. 10.1093/gigascience/giz101 PMC6701489 31430367

[B17] Kang Z.-W. Tian H.-G. Liu F.-H. Liu X. Jing X.-F. Liu T.-X. (2017). Identification and Expression Analysis of Chemosensory Receptor Genes in an Aphid Endoparasitoid *Aphidius Gifuensis* . Sci. Rep. 7 (1), 3939. 10.1038/s41598-017-03988-z 28638084 PMC5479799

[B18] Leal W. S. (2013). Odorant Reception in Insects: Roles of Receptors, Binding Proteins, and Degrading Enzymes. Annu. Rev. Entomol. 58, 373–391. 10.1146/annurev-ento-120811-153635 23020622

[B19] Li M.-Y. Jiang X.-Y. Qi Y.-Z. Huang Y.-J. Li S.-G. Liu S. (2020). Identification and Expression Profiles of 14 Odorant-Binding Protein Genes from *Pieris Rapae* (Lepidoptera: Pieridae). J. Insect Sci. 20 (5), 2. 10.1093/jisesa/ieaa087 PMC7474526 32889524

[B20] Li S. Picimbon J.-F. Ji S. Kan Y. Chuanling Q. Zhou J.-J. (2008). Multiple Functions of an Odorant-Binding Protein in the Mosquito *Aedes aegypti* . Biochem. Biophysical Res. Commun. 372 (3), 464–468. 10.1016/j.bbrc.2008.05.064 18502197

[B21] Li T.-T. Liu W.-C. Zhu J. Yang Y.-H. Ma C. Lu C. (2019). Crystal Structure and Ligand Identification of Odorant Binding Protein 4 in the Natural Predator *Chrysopa Pallens* . Int. J. Biol. Macromolecules 141, 1004–1012. 10.1016/j.ijbiomac.2019.09.043 31525411

[B22] Li Y.-j. Chen H.-c. Hong T.-l. Yan M.-w. Wang J. Shao Z.-m. (2021). Identification of Chemosensory Genes by Antennal Transcriptome Analysis and Expression Profiles of Odorant-Binding Proteins in Parasitoid Wasp *Aulacocentrum Confusum* . Comp. Biochem. Physiol. D: Genomics Proteomics 40, 100881. 10.1016/j.cbd.2021.100881 34273642

[B23] Li Z.-Q. Zhang S. Cai X.-M. Luo J.-Y. Dong S.-L. Cui J.-J. (2017). Three Odorant Binding Proteins May Regulate the Behavioural Response of Chrysopa Pallens to Plant Volatiles and the Aphid Alarm Pheromone (E)-β-farnesene. Insect Mol. Biol. 26 (3), 255–265. 10.1111/imb.12295 28247518

[B24] Liu N.-Y. Zhang T. Ye Z.-F. Li F. Dong S.-L. (2015). Identification and Characterization of Candidate Chemosensory Gene Families from *Spodoptera Exigua* Developmental Transcriptomes. Int. J. Biol. Sci. 11 (9), 1036–1048. 10.7150/ijbs.12020 26221071 PMC4515815

[B25] Liu Y. Du L. Zhu Y. Yang S. Zhou Q. Wang G. (2020). Identification and Sex-Biased Profiles of Candidate Olfactory Genes in the Antennal Transcriptome of the Parasitoid Wasp *Cotesia Vestalis* . Comp. Biochem. Physiol. Part D: Genomics Proteomics 34, 100657. 10.1016/j.cbd.2020.100657 32032903

[B26] Livak K. J. Schmittgen T. D. (2001). Analysis of Relative Gene Expression Data Using Real-Time Quantitative PCR and the 2−ΔΔCT Method. Methods 25 (4), 402–408. 10.1006/meth.2001.1262.Prestwich 11846609

[B27] Ohta I. Honda K.-i. (2010). Use of *Sitobion Akebiae* (Hemiptera: Aphididae) as an Alternative Host Aphid for a Banker-Plant System Using an Indigenous Parasitoid, *Aphidius Gifuensis* (Hymenoptera: Braconidae). Appl. Entomol. Zool. 45 (2), 233–238. 10.1303/aez.2010.233

[B28] Pelosi P. Calvello M. Ban L. (2005). Diversity of Odorant-Binding Proteins and Chemosensory Proteins in Insects. Chem. Senses 30 (Suppl. 1), i291–i292. 10.1093/chemse/bjh229 15738163

[B29] Pelosi P. Iovinella I. Felicioli A. Dani F. R. (2014). Soluble Proteins of Chemical Communication: an Overview across Arthropods. Front. Physiol. 5, 320. 10.3389/fphys.2014.00320 25221516 PMC4145409

[B30] Pelosi P. Iovinella I. Zhu J. Wang G. Dani F. R. (2018). Beyond Chemoreception: Diverse Tasks of Soluble Olfactory Proteins in Insects. Biol. Rev. 93 (1), 184–200. 10.1111/brv.12339 28480618

[B31] Pelosi P. Zhou J.-J. Ban L. P. Calvello M. (2006). Soluble Proteins in Insect Chemical Communication. Cell. Mol. Life Sci. 63 (14), 1658–1676. 10.1007/s00018-005-5607-0 16786224 PMC11136032

[B32] Peng Y. Wang S.-N. Li K.-M. Liu J.-T. Zheng Y. Shan S. (2017). Identification of Odorant Binding Proteins and Chemosensory Proteins in *Microplitis Mediator* as Well as Functional Characterization of Chemosensory Protein 3. PLoS One 12 (7), e0180775. 10.1371/journal.pone.0180775 28732030 PMC5521769

[B33] Powell W. Pennacchio F. Poppy G. M. Tremblay E. (1998). Strategies Involved in the Location of Hosts by the ParasitoidAphidius erviHaliday (Hymenoptera: Braconidae: Aphidiinae). Biol. Control. 11, 104–112. 10.1006/bcon.1997.0584

[B34] Prestwich G. D. (1993). Bacterial Expression and Photoaffinity Labeling of a Pheromone Binding Protein. Protein Sci. 2, 420–428. 10.1002/pro.5560020314 8453379 PMC2142376

[B35] Qiao H. Tuccori E. He X. Gazzano A. Field L. Zhou J.-J. (2009). Discrimination of Alarm Pheromone (E)-β-farnesene by Aphid Odorant-Binding Proteins. Insect Biochem. Mol. Biol. 39 (5-6), 414–419. 10.1016/j.ibmb.2009.03.004 19328854

[B36] Qin Y. G. Yang Z. K. Song D. L. Wang Q. Gu S. H. Li W. H. (2020). Bioactivities of Synthetic Salicylate‐substituted Carboxyl ( E )‐β‐Farnesene Derivatives as Ecofriendly Agrochemicals and Their Binding Mechanism with Potential Targets in Aphid Olfactory System. Pest Manag. Sci. 76 (7), 2465–2472. 10.1002/ps.5787 32061021

[B37] Sheng S. Liao C.-W. Zheng Y. Zhou Y. Xu Y. Song W.-M. (2017). Candidate Chemosensory Genes Identified in the Endoparasitoid *Meteorus Pulchricornis* (Hymenoptera: Braconidae) by Antennal Transcriptome Analysis. Comp. Biochem. Physiol. Part D: Genomics Proteomics 22, 20–31. 10.1016/j.cbd.2017.01.002 28187311

[B38] Song X. Qin Y.-G. Yin Y. Li Z.-X. (2021a). Identification and Behavioral Assays of Alarm Pheromone in the Vetch Aphid *Megoura Viciae* . J. Chem. Ecol. 47 (8-9), 740–746. 10.1007/s10886-021-01297-4 34347235

[B39] Song Y. Liu C. Cai P. Chen W. Guo Y. Lin J. (2021b). Host-Seeking Behavior of *Aphidius Gifuensis* (Hymenoptera: Braconidae) Modulated by Chemical Cues within a Tritrophic Context. J. Insect Sci. 21 (3), 9. 10.1093/jisesa/ieab036 PMC8161523 34047335

[B40] Sun L. Wei Y. Zhang D.-D. Ma X.-Y. Xiao Y. Zhang Y.-N. (2016). The Mouthparts Enriched Odorant Binding Protein 11 of the Alfalfa Plant Bug *Adelphocoris Lineolatus* Displays a Preferential Binding Behavior to Host Plant Secondary Metabolites. Front. Physiol. 7, 201. 10.3389/fphys.2016.00201 27313540 PMC4887496

[B41] Sun Y.-L. Huang L.-Q. Pelosi P. Wang C.-Z. (2012b). Expression in Antennae and Reproductive Organs Suggests a Dual Role of an Odorant-Binding Protein in Two Sibling Helicoverpa Species. Plos One 7 (1), e30040. 10.1371/journal.pone.0030040 22291900 PMC3264552

[B42] Sun Y. F. De Biasio F. Qiao H. L. Iovinella I. Yang S. X. Ling Y. (2012a). Two Odorant-Binding Proteins Mediate the Behavioural Response of Aphids to the Alarm Pheromone (E)-ß-farnesene and Structural Analogues. PLoS One 7 (3), e32759. 10.1371/journal.pone.0032759 22427877 PMC3299684

[B43] Tamura K. Stecher G. Kumar S. (2021). MEGA11: Molecular Evolutionary Genetics Analysis Version 11. Mol. Biol. Evol. 38 (7), 3022–3027. 10.1093/molbev/msab120 33892491 PMC8233496

[B44] Vieira F. G. Forêt S. He X. Rozas J. Field L. M. Zhou J.-J. (2012). Unique Features of Odorant-Binding Proteins of the Parasitoid Wasp *Nasonia vitripennis* Revealed by Genome Annotation and Comparative Analyses. PLoS One 7 (8), e43034. 10.1371/journal.pone.0043034 22952629 PMC3428353

[B45] Vogt R. G. Riddiford L. M. (1981). Pheromone Binding and Inactivation by Moth Antennae. Nature 293 (5828), 161–163. 10.1038/293161a0 18074618

[B46] Wang B. Dong W. Li H. D’Onofrio C. Bai P. Chen R. (2022). Molecular Basis of (E)-β-farnesene-mediated Aphid Location in the Predator Eupeodes Corollae. Curr. Biol. 32 (21), 951–962. 10.1016/j.cub.2021.12.054 35065682

[B47] Wang L. Bi Y. D. Liu M. Li W. Liu M. Di S. F. (2019). Identification and Expression Profiles Analysis of Odorant‐binding Proteins in Soybean Aphid, Aphis Glycines (Hemiptera: Aphididae). Insect Sci. 27, 1019–1030. 10.1111/1744-7917.1270910.1111/1744-7917.12709 31271503

[B48] Wang L. Yin H. Zhu Z. Yang S. Fan J. (2021a). A Detailed Spatial Expression Analysis of Wing Phenotypes Reveals Novel Patterns of Odorant Binding Proteins in the Soybean Aphid, *Aphis Glycines* . Front. Physiol. 12, 702973. 10.3389/fphys.2021.702973 34421640 PMC8376974

[B49] Wang Q. Liu J.-T. Zhang Y.-J. Chen J.-L. Li X.-C. Liang P. (2021b). Coordinative Mediation of the Response to Alarm Pheromones by Three Odorant Binding Proteins in the green Peach Aphid *Myzus persicae* . Insect Biochem. Mol. Biol. 130, 103528. 10.1016/j.ibmb.2021.103528 33482303

[B50] Wu J. X. (2002). Agricultural Entomology. Beijing: China Agriculture Press.

[B51] Xu X. Xu W. Ishida Y. Li Y. Leal W. S. Ames J. B. (2009). 1H, 15N, and 13C Chemical Shift Assignments of the Mosquito Odorant Binding Protein-1 (CquiOBP1) Bound to the Mosquito Oviposition Pheromone. Biomol. NMR Assign 3 (2), 195–197. 10.1007/s12104-009-9173-5 19888689 PMC2772962

[B52] Xue W. Fan J. Zhang Y. Xu Q. Han Z. Sun J. (2016). Identification and Expression Analysis of Candidate Odorant-Binding Protein and Chemosensory Protein Genes by Antennal Transcriptome of *Sitobion Avenae* . PLoS One 11 (8), e0161839. 10.1371/journal.pone.0161839 27561107 PMC4999175

[B53] Yang S. Xu R. Yang S.-Y. Kuang R.-P. (2009). Olfactory Responses of Aphidius *gifuensis* to Odors of Host Plants and Aphid-Plant Complexes. Insect Sci. 16 (6), 503–510. 10.1111/j.1744-7917.2009.01282.x

[B63] Yang S. Wei J. Yang S. Kuang R. (2011). Current Status and Future Trends of Augmentative Release of Aphidius Gifuensis for Control of Myzus Persicae in China Yunnan Province. Journal of the Entomological Research Society 13 (3), 87–99.

[B54] Yasukawa J. Tomioka S. Aigaki T. Matsuo T. (2010). Evolution of expression patterns of two odorant-binding protein genes, obp57d and obp57e, in drosophila. Gene 467 (1-2), 25–34. 10.1016/j.gene.2010.07.006 20637846

[B55] Yuan H.-B. Ding Y.-X. Gu S.-H. Sun L. Zhu X.-Q. Liu H.-W. (2015). Molecular Characterization and Expression Profiling of Odorant-Binding Proteins in *Apolygus Lucorum* . PLoS ONE 10 (10), e0140562. 10.1371/journal.pone.0140562 26466366 PMC4605488

[B56] Zhang G. (1999). Aphids in Agriculture and Forestry of Northwest China. 1st ed. Beijing: China Environmental Science.

[B57] Zhang S. Zhang Y.-J. Su H.-H. Gao X.-W. Guo Y.-Y. (2009). Identification and Expression Pattern of Putative Odorant-Binding Proteins and Chemosensory Proteins in Antennae of the *Microplitis Mediator* (Hymenoptera: Braconidae). Chem. Senses 34 (6), 503–512. 10.1093/chemse/bjp027 19497961

[B58] Zhang Y. Fan J. Sun J. Francis F. Chen J. (2017). Transcriptome Analysis of the Salivary Glands of the Grain Aphid, Sitobion Avenae. Sci. Rep. 7 (1), 15911. 10.1038/s41598-017-16092-z 29162876 PMC5698471

[B59] Zhong T. Yin J. Deng S. Li K. Cao Y. (2012). Fluorescence Competition Assay for the Assessment of green Leaf Volatiles and Trans-β-farnesene Bound to Three Odorant-Binding Proteins in the Wheat Aphid Sitobion Avenae (Fabricius). J. Insect Physiol. 58 (6), 771–781. 10.1016/j.jinsphys.2012.01.011 22306433

[B60] Zhou C.-X. Min S.-F. Yan-Long T. Wang M.-Q. (2015). Analysis of Antennal Transcriptome and Odorant Binding Protein Expression Profiles of the Recently Identified Parasitoid Wasp, Sclerodermus Sp. Comp. Biochem. Physiol. Part D: Genomics Proteomics 16, 10–19. 10.1016/j.cbd.2015.06.003 26164593

[B61] Zhou J.-J. Vieira F. G. He X.-L. Smadja C. Liu R. Rozas J. (2010). Genome Annotation and Comparative Analyses of the Odorant-Binding Proteins and Chemosensory Proteins in the Pea Aphid *Acyrthosiphon pisum* . Insect Mol. Biol. 19 (Suppl. 2), 113–122. 10.1111/j.1365-2583.2009.00919.x 20482644

[B62] Zhou Y. N. Xie S. Chen J. N. Wang Z. H. Yang P. Zhou S. C. (2021). Expression and Functional Characterization of Odorant-Binding Protein Genes in the Endoparasitic Wasp Cotesia Vestalis. Insect Sci. 28 (5), 1354–1368. 10.1111/1744-7917.12861 32761881

