# Supplementary material for: Spatial Expression Analysis of Odorant Binding Proteins in Both Sexes of the Aphid Parasitoid Aphidius gifuensis and Their Ligand Binding Properties
Source: Front Physiol. 2022 May 4;13:877133. doi: 10.3389/fphys.2022.877133 (PMC9115719; doi:10.3389/fphys.2022.877133)
Supplement: Supplementary file 1 [file Table1.DOCX]

| *OBP1* | F | CGGGATCCAATGAATATTTTTCAAAATTCATTG |
| --- | --- | --- |
|  | R | CCGCTCGAG TTATGAACCAAGAAGTAGTGCAGGA |
| *OBP2* | F | CCCATATGCGTCCCAGTTTTGTATCAGATGA |
|  | R | CGGAATTCTTAAAACAGGTAATATGTTTTTGGT |
| *OBP3* | F | CCCATATGAAATTGCCGGATTTTATAACAC |
|  | R | CGGAATTCTCATATCATAAACCACATATCAGGT |
| *OBP4* | F | CCCATATGGCATTGACTCCTGAGCAAAATTCTA |
|  | R | CGGAATTCTTAAGCTCCAATGACTTTGTTGTCT |
| *OBP5* | F | CCCATATGCAGGATCCAGATTGTCCAGTTTAT |
|  | R | CGGAATTCTTAGGCTCCAGCTTTTTCTGCTTTG |
| *OBP6* | F | CCCATATGAAAATGACTTTGGCCCAAGTTA |
|  | R | CGGGATCCTTAAGGCAAAAAATACATATCTGCA |
| *OBP7* | F | CCCATATGAATCATGAAAAATTTCATGAAGC |
|  | R | GCGAATTCTTAGTTACCATCTGGTTTTTGGG |
| *OBP8* | F | CCCATATGGATGATCCTCATGCATCAATCAG |
|  | R | CGGAATTCTCATATAGTTGGTGGACGAAAAATT |
| *OBP9* | F | CCCATATGCTTATTAGGCTTCAAGCTGCTAA |
|  | R | GCGAATTCTCAGCCTAAAGCTTGTACTGGAA |
| *OBP11* | F | CCCATATGTTTTTTGTTTTTGCTGGTGAAATTC |
|  | R | GCGAATTCTTATATGATGAAAAATTCTACTGGA |
| *OBP12* | F | CCCATATGCTGAGATGTCGTTCAGGTAATCA |
|  | R | CGGGATCCTTAGTAGTTTTCGTCCCAATCTTCA |
| *OBP13* | F | CCCATATGATCATGGAAGATCTTGCAATC |
|  | R | CGGGATCCCTATTCGGTATCTTTTTTTCTAATT |
| *OBP15* | F | CCCATATGTCTGCTGGTCCAGTACCAAAAG |
|  | R | GCGAATTCTTACATGATGAAATAATCATCAGG |
| *OBP17* | F | GTTCGACCTCCAATTCTT |
|  | R | TCAGCAACTTTCCCTTCT |

Table 3. Primer lists for RT-PCR

Table 4. Primer lists for qRT-PCR.

| Gene names | direction | Primer Sequences （5'→3'） |
| --- | --- | --- |
| OBP1 | F | TACAGATGCTGCTATTTC |
|  | R | CACTTATCTTCTCCCTTT |
| OBP2 | F | TTGCACTTATTGATGACAGA |
|  | R | TTTTGGTGATGATTTTGC |
| OBP3 | F | GTTGGAATGTTCCCAGAA |
|  | R | CGCAGTCATCAGAGCC |
| OBP4 | F | GCTTGTATTGTTGGTGCTTT |
|  | R | GACTCATCCCAATTTCCTTT |
| OBP5 | F | AAATCGCCAACAACAG |
|  | R | AGCCACTTCGCACTC |
| OBP6 | F | AAAGCATAACCGACAA |
|  | R | TCGGCTTCATAATAACAC |
| OBP7 | F | AAAGTGTTGGGGAGC |
|  | R | GATCATTTCGATAGCCT |
| OBP8 | F | GTGGCTTACTTGTCTTATC |
|  | R | AATTCAGCTCATTTCG |
| OBP9 | F | AAATCGTGCTCGTCA |
|  | R | AAGTCAGTCCCAGTTGT |
| OBP11 | F | AGGATTAGTAACCGATGAA |
|  | R | GCTATCAGTTCCCTTTGT |
| OBP12 | F | GAATGCGAAACGAAA |
|  | R | GTGCCTGTTACTGCTGA |
| OBP13 | F | GATCATCATGGCAGTTT |
|  | R | CGAGTACAAGGGTCTG |
| OBP15 | F | GAAGCTGGACTTGGA |
|  | R | TTTGGTGGAGATAACG |
| OBP17 | F | AAAATGACTGAGGCTTGT |
|  | R | TTAATGTCGCTAATTCTGA |
| Actin | F | ACAGCAGCATCATCATCAA |
|  | R | TCTGGACAACGGAATCTTT |
| NADH | F | CTGGCACTGGGATAAAAC |
|  | R | TCAGGAATTGGTGAAAGC |
