# Supplementary material for: Spatial Expression Analysis of Odorant Binding Proteins in Both Sexes of the Aphid Parasitoid Aphidius gifuensis and Their Ligand Binding Properties
Source: Front Physiol. 2022 May 4;13:877133. doi: 10.3389/fphys.2022.877133 (PMC9115719; doi:10.3389/fphys.2022.877133)
Supplement: Supplementary file 2 [file DataSheet2.docx]

>AgifOBP1

MKHIFFLIIIFTFSLSIEAADNEYFSKFIAATQQCMENNKVDDSILSRVLEGEMVDDKSFDCFVACLLEKLELIGSDGSLNTDAAISKIPADIKIHDQLEKVVRTCSTRKGEDKCSTAHMLFVCLHENDVPALLLGS-

>AgifOBP2

MFINKQRTTMRNLVITMILIFQISFIYCESRPSFVSDDMIAAAASVVNACQTQTGVATADIEAVRNGDWPDSEPLKCYMNCMMESFALIDDRKEISLNGMLSFFQRIPAYREEVEKTVRKCKYIGKHLANGDNCQYAYTFNLCYAKSSPKTYYLF-

>AgifOBP3

MENFIVKYIFFGILLQAVFITAKLPDFITPDMVAMVADDKAKCMGLHGTTEALIDQVNEGTIVNDRAITCYMHCLFETFGVIDEDGELEVEMLVGMFPESIQDAGRELFNKCASQTGSDDCDKVFNIAKCVQQTRPDMWFMI-

>AgifOBP4

MKFFAIIFVACIVGAFGALTPEQNSKLEEIRAACAKESSADPAKIENAKKGNWDESDPKLGQFSSCFLKKLGLMDNSGNLNVELTREKIGKVVSAEKADEIMKKCKDLKGDNADQTGIKLLKCYTDNKVIGA-

>AgifOBP5

MKYLAIVGLIGLIFFVSNGLSQDPDCPVYKLMMASVEKCKGQLSEENAKLMEKNPGVENDEINCFRGCVLVGMGVMKNAKIDIENLKELMKQSKSPTTAEAVVTVARECKKQSEVSNNECEVAGSYTKCVVALKDKAEKAGA-

>AgifOBP6

MMLISIVGFTIFLVVSIDINNVEAKMTLAQVRNSLKPFHKACLPKSGVSPDVWEATHNGEFPPDPALQCHFACLFTKLKILTKDGKLSMESMAKQMDIMLPEDLVGPIKSITDKCAVDATSSEVCEMSWQFAKCYYEADADMYFLP-

>AgifOBP7

MNTSSVILVFCALAITMVVGNHEKFHEAIAKCKEELSIDDEMFENHKKNHFISEDPKLKCWGACLMKKMGTMTNEGVVMKEKAIEMIPADMKNRDKLMEAIETCSIKSGADECETASMVHKCIKEKMPERPQKPDGN-

>AgifOBP8

MKISGLLVLSIVLFVYGDDPHASIRANCRNELNLTDQELIDAIPDPINMDCYLYCFLMDINVMDIKGNFNPAAAVQSIQDELKDAAKPNIYACYEQTKENMDEEPCTRAYDVIKCFQTRSPDLYEKLGIFRPPTI-

>AgifOBP9

MKFIFLFLTFAILAYNVKAQTAAGLIRLQAANRLCRQQNGIDRSLINRARQGEFIDNNPQFDCYVGCLLQQLGLTYDDGSLDVNTAVNMVPLTSPSHDQIVNAISICGNQRGNDKCSTAHSLYSCMYQNNIPVQALG-

>AgifOBP11

MNKKIISICFFNFLYVFFVFAGEIPEEFQSFSKDLRAYCIEKSGVNGELIELAVKGLFVKDRKLGCYSYCVAQQLGLVTDEKMDFKKFLILTPPRLKEKSKVLVSSCKDTKGTDSCDLAYNINYCFYKTYPVEFFII-

>AgifOBP13

MDKLIGLSLFFTLVSSSAIMEDLAIVRICNATDSVDISILNDYMLNHDFHTLENHQLRQLSCFLLCIYSEYNWMDHHGSFKIHNIKSWMHRAKLPTDHIEILLKRCITSELTDPCTRARHFTECFWSNHQGILNANHRHTLHSIIRKKDTE-

>AgifOBP14

MQTKADIRRECRKQTGVAWDPLSKFKNGDFNENDPKLKCYLKCFMQKYGIFGDDSIYIDRVLRYLPYSMQKTSKNTLEKCNLIPSTDSCDKAFQLLKCYFKSQPEVIFLKLLYYFTV-

>AgifOBP15

MTKAMLIVLFFTSLVIYTSAGPVPKEFEDVAPEIRKICLAESGTTNEMVNEVGLGKFTEDDKLKCYLRCLFDQFRLMTPKGLNFKGFLALSPPNMKEKAVIMVEKCKETTGKDLCELSFNLHKCFYEAFPDDYFIM-

>AgifOBP17

MINVKFLFTLGIIFIIIAICFSESFFSQCVRPPILHESVYACMDSLDNEGKEFLKNETNHNSPKLTAFKVCLLVQFNFLKNGTIREQQQELFIKKYVEDKKAAIKMTEACQICRDNANAQNEEGKVAEYFFNCLKENSELATLISDKLCIKQNNDNTTSKKLSSK-

>AgifOBP19

HMLRCRSGNQQISNEFRRTMQKCKNHYSGSSRTGDDNFSSSNNDNSSDEDSNSDEILFEHDFFTGSRKNNSQSMGRDEMRNNQRNDRNNRNNFSNSRNNSNMGSNRMSNGNSNNNWNAKRNNRDMEDDDNSNNGHSGQSCSIQCFFNELNLVDQRGYPERSAVTGTLMRGVQDPMLRDFIEESIMECFHFVSSIMNQDKCKFSERLFTCFAEKGREGCEDWDENY-

>MmedOBP1

MKNIIIFTTAITIFTFINFSQTEARMTMTQIRNAMKPLGKTCLGKTGLSKEVQAGQHNGEFPEDEALMCYHSCLLKLAKISDKSGNINLDTVHKQIDLMMPEDLIARAKAVTTDCFGEIKSTEICRMSFEFVKCYFIKGPEIVFFP-

>MmedOBP2

MKSIIFLGVLLTVLISNKAEAKSVQKRECPFKKPFEANAPKCMDKISEENMGRMMQGNMDNDEIRCFVGCVFENAGFVKDNKVQMDKVREAVDNFVDDYKYSKEVGDQVYGVVSDCAPQAEKGANNCEVSSNLLICFKTNNKFT-

>MmedOBP3

MRGSVLAIVACALVVGVLGDDDMKEKHKEIFKKCAEETGVTKEDLHNHKRGEEPETKIKCFHACIAKADGAMVDGKLNKDKVIEKIPADLPDRERIIEAVTKCSEQTAADECETAHLVFKCLRENKALPHPPHHHHHHHDE-

>MmedOBP4

MKCFTLTAAGILFTVLITVNNASSNSNMEELVKKSMEETFKACKDKLTPENFALLNKDPHADNQEIKCFKACGMNHAGIMADGKIQIEKMEEKLNSLLGEDKKDFSKIIIGRAKPCVEEANKGENECDVAAGFEACVQKTINTKSDN-

>MmedOBP5

MKNFVVIVILALYFTATTESLQEIMNTFQKARLEVRAPCLHLLSNETLTTLKTRRHLDNPEIRCFKACLMERQGYLKDNKIFIDEYEKLIDVNLKRIKELNMKFARACVNEAEKSENKCELAHNYNRCILHQTRKHYNQTAEENDENQNQHL-

>MmedOBP6

MKNTLFFTLAAAFLLGYNIPHVESRMSMAQTINTMKPLGKTCAAKTGLSKEMQDGQHEGQFPEEEALMCYHTCLLKMAKVADKTGKLNIDAMVKQIDMLMPEDLVDKAKTACSGCADEVTATEGCRPSWEFMKCWYGRAPELYFFP-

>MmedOBP8

MDSNIKYMCFLYIFIVVFMFFSEAIDQSDPHASTRKKCSGEFKLTDEILKLGEQDPSDFSCYLFCLFKDINIMNQKGEFDPNLAAQEVQDNLREAARKYIFMCYDLVKPNMTSDGCKNALEMVQCFKEKAPEMYEMLGLFHPPSNEPLKMTQ-

>MmedOBP10

MAKFLLSSVGVLVLIAYVQSGPVPEEFKDVQPTIRAACVKESGLTNEELVNKAALGEFTDDPQLKCYLKCIFDQFRLVSKRGINFDAMLALSPPSMKENAIKMVKECRDTKGKEGDLCDLSFEVTKCLYNSNPETYFIL-

>MmedOBP11

MKIFAVIFAICIVYAVAIGNLTEEERVELDRLANICVNETGFYEGHNSDDPAKNWISYGFKLQCYFSCMLKKMNIMNEDGTLNEEMIRKKIGDEVPADKIDAVITKCKDLKGANKCETATMIMKCYSDERLSLDPAEKSV-

>MmedOBP12

MAIVRICNTTDPVDLRVLNDYLMNHNLNRLHIKSHHPLACFLLCVYSEFNWMDRHGGFKVHNIKAWMLRAELSENDTDILLRKCISLELTDPCTRAQYFTECFWTNHQDVTVDHRHSLHSIMHKDVHQDKIYN-

>MmedOBP13

MKIIAVIFAVCFAGALAELTVEQLAKLREHSTACITETLVDDANVDAAMHHNIWRMDDLKLRCYFFCLLKKLKVMNEDGKLNEEITRQRLANLFPADRIDGVIMKCKEMKGADACETAILMAKCHADERGLLGPSPRSA-

>MmedOBP14

MKGVKSIPLIAIAAVFCISINFFSTDAAFTVEQIESMMKPLGNNCVSKVGLSPELQEANRKKEFPEEKPFMCYLHCLARVTKVFDKNNQIDLEGTLKQVRLVMPDHLVEGSVKAYTVCSRAAISEDPCEKAFQYAKCYYETDAPSYFYP-

>MmedOBP15

MKNILLGICIFIPSVFCGTRPSFVSDDVIGFAASGVNACQRQTGVATADIEAVRNGQWPESRQLKCYMYCLWEQFGLIDEKGELSLNGMLTFFQRIPAYRVEVQKAIRECKSIGEYLANGDNCQYAFTFNMCYAEVSPKTYYLF-

>MmedOBP16

MKLFAVLFAVCFVGALAELTPEQLAKLHESRSTCITETGVEEGNVAKANDGEWLMDDLKLRCFFSCMLKKIKVLNEDGTFNEEKARKRIANDLPADKIDSVITKCKDLSGGDVCETAMLMMKCYADEKALTKIITEKSS-

>MmedOBP17

MKIFAVIFAVCFVAALAELTEEQKAKLREHRTACVTETGVDEANVDAAKQGDWKMDDLKLRCFFACMMKKIKVMNEDGTLNEEITRKRMANDLPADKIDGVMMKCKDMKGADMCETAMMMMKCYADEKAFTKIITEKSS-

>MmedOBP18

MRYSVFVFVGILFTFFISSDAESSGEKECPLKKAFQESIDACKDKLSEENLALLEKDENADNEDIRCFKACILNDSGVMSNGKIQIDKIEEAINAAIENVKEDEEKAKAIGESMINGAKNCAGPAEEGENECEVAHRFITCLMEHAAEEKKKHNE-

>MmedOBP19

MYRLAVVFIFASVVVLSESAITAEDLVKFGMARRTCDRTNRVDPSVIDRVLQGEMINDPQFDCHVACVLKELNLLTADGSLNVEVAASKVPENLPYYNQLVGAIRSCGSRKGNDQCETAHMLFVCFHENNIPNLILG-

>MmedOBP20

MQMQVNADIKRDCRKQTGVSWASLKKLKAADYNQNDPKLKCYLKCFMQKNGIFGEDDIDIEKALRHLPTGIKGPSKTTLEYCKKIPSVDSCDKAFQLAKCYFKAQPEVLKSVSFV-

>MpulOBP1

MKNIGSASSCLVLFFVICVNANGIVEHKALREKCRDESKLTDDDIKMSTMIADHLGCYLFCFLKDLEVMDDKGSFDPAAATDAVEEELREASRPEIYSCYESYSSDDDALNDNACSTALEMTRCFKEHAQNLYEVMGIFEPVV-

>MpulOBP2

MQYLTTLLLASIAFAVVTALTPQDIINARMTIQRCNNGNVDPSLITQALGGQMVNDREFDCFIACILEGIRVSNADGSLNVDNALSKLPQNIASRDIIVDAIKSCGDQRGDDKCETAHMLYQCMQEKNIPTTTLLG-

>MpulOBP3

MKTIAVVFAVCIVGALGTLTEEHKAKLREHRDKCIDETKVDRTLVDKAHGGQWQEGDEKLQCFAACLLKKLGMMAEDGKLNEEVSLAKMTLDVGAEKAREIWDNCKDKTGANTCAKGFELMKCYTSKKTLLLA-

>MpulOBP4

MKTIAVVFAVCIVGALGNLTEEHKAKLREHRDKCIRETKVDRTLVDKAHGGQWQEGDEKLQCFAACLLKKLGMMAEDGKLNEEVSLAKMTLDVGAEVAREIWDNCKDKTGANTCAKGFELMKCYTSKKTLLLA-

>MpulOBP5

MSRFVFNCVVHGILMPTFLVSAKLPDWVSDDMIEMVGEDKARCLGETGATQGLIDEVNEGKLPTDKSLACYMYCLFESLSLVDEDGVLDYEMIAGSLPDDMQSTATNILGACAAQPGADNCEKMYNIGVCVQAKDPSMFFML-

>MpulOBP6

MNNNMQKNRVRYISSAAFLTIAILVAINIQDGEAGATIAQIRESMKPIGDACIPETGVCEEMLAKTQQGEFPADPELQCYYACVFRMMEVMNGNDQIDTDMVMDKIDAMLPEDLAQRVKENSKICFSKITSNDLCVMSWEFTKCFYELDSSLFFFP-

>MpulOBP7

MKSFLISTLAILLSVATIVHSGPIPDEFKDVAPDIRETCTKLTSVSTDLIEKAGLGDFVEDDKLKCYLKCLFDQFRLLSPKGFNFEAMLGLTPPKMKDAAVKAVKDCRDTTGKPDDMCDLSFNLHKCFYNSSPDQYFIM-

>MpulOBP8

MMITNVWICLMLVLQCNYAMRCRSGNSEVVNDIEKVILQCRRSDSQSGRNDSDSSRLNDSTEDDFASSDSSGLFNQDFFSNAKKDIQNITNNYGNGSSFKGNNPDSYGQQSSIQSSYMNPSNYTNMREYQNQKTNHNDNGFMIDGMQQENQGTDGDPRQSCIVQCIFEELNSVDQRGFPERASVTRMLLRGIQDPMVHDFIGESILQCFQFLSSEMNYDKCTFSQNLLNCLADKGKEQCEDWND-

>MpulOBP9

MSRFVFNCVVIGILMPTFLVSAKLPDWVSDDMIEMVAEDKARCLGETGATQGLIDEVNEGKLPTDKSLACYMYCLFESLSLVDEDGVLDYEMIAGFLPDDMQSTATNILGACAAQPGADNCEKMYNIGVCVQAKDPSMFFML-

>MpulOBP10

MNNNMQKNRVRYISSAAFLTIAILVAINIQDGEAGATIAQIRESMKPIGDACIPETGVCEEMLAKTQQGEFPADPELQCYYACVFRMMEVMNGNDQIDTDMVMDKIDAMLPEDLAQRVKENSKICFSKITSNDLCVMSWEFTKCFYELDSSLFFFP-

>MpulOBP11

MYRSTITILVLCALSIGVLTHHHGPPPEVKAAMDKCVKEAGGDESTMPNLRRHEELADPKFKCVAACVMKELGQMSADGTVDKNSAFAKMPEDIPDRDKLIAEMGPCFDEKGADECETANLIRKCMMEKMPRGPPPH-

>MpulOBP12

MKSFLISTLAILLSVATIVHSGPIPDEFKDVAPDIRETCTKLTSVSTDLIEKAGLGDFVEDDKLKCYLKCLFDQFRLLSPHGFNFEAMLGLTPPKMTDAAVKAVKDCRDTTAKPDDMCDLSFNLHKCFYNSSPDQYFIM-

>MpulOBP13

MRNLVVVFFLIQSSFVLCGSRPSFVTDAMIAVAASVVNACQTQTGVATADIEAVRNGTWPDSRPLKCYMYCLWVQFGLVDENDELSVNGMLTFFQRMPAYRTEVSIALRECNGIGKYLAHGDNCTYAYTFNMCYAMLSPKTYYLF-

>MpulOBP14

MKNTTSFIFIGILCSVFLASNGEKECPMKEALAESIEACKDKISEDSAKLLKADETADNEEIRAFKACVMIHGGILEDQKIKIDKIQEMLKNHVEEEEMENVMTVMKICQGEAEIGADDGEVATLFINCFKKATADSNAAK-

>MpulOBP15

MGLIPCSICLITFVFLAINIHNSEAKMTLPQVRNALKPGAKTCMTKTGVSKSLVEKTHEGEFPTDPALQCYFACILKLMKVVSKDEHIDLDMMHKQADLLMVQNLANQVKQLTQTCYENITSSEVCEMSWELVKCYHELDSSMYFFP-

>MpulOBP16

MKNNGMRYISPVALFTIVFLVAINIHDSEAKMSMAQIKNMMKPVSKTCITKIGVSKDLIDKTHEGEFPPDPQLQCYYACIFKMMKVVTKDEQVDLNLILKQINMLALEELGKQITPIVQDCDAKITATEVCEVSWAFAKCLWETDQSMYFFP-

>AmelOBP2

MNTLVTVTCLLAALTVVRGIDQDTVVAKYMEYLMPDIMPCADELHISEDIATNIQAAKNGADMSQLGCLKACVMKRIEMLKGTELYVEPVYKMIEVVHAGNADDIQLVKGIANECIENAKGETDECNIGNKYTDCYIEKLFS

>AmelOBP1

MASNTKQAFIYSLALLCLHAIFVNAAPDWVPPEVFDLVAEDKARCMSEHGTTQAQIDDVDKGNLVNEPSITCYMYCLLEAFSLVDDEANVDEDIMLGLLPDQLQERAQSVMGKCLPTSGSDNCNKIYNLAKCVQESAPDVWFVI

>AmelOBP6

MKGLGVSLLVALLLVLLAIEDTMSKKMTIEEAKKTIKNLRKVCSKKNDTPKELLDGQFRGEFPQDERLMCYMKCIMIATKAMKNDVILWDFFVKNARMILLEEYIPRVESVVETCKKEVTSTEGCEVAWQFGKCIYENDKELYLAP

>AmelOBP5

MHVKSVLLLITIVTFVALKPVKSMSADQVEKLAKNMRKSCLQKIAITEELVDGMRRGEFPDDHDLQCYTTCIMKLLRTFKNGNFDFDMIVKQLEITMPPEEVVIGKEIVAVCRNEEYTGDDCQKTYQYVQCHYKQNPEKFFFP

>AmelOBP14

MKTIVLIFGFCVCVGALTIEELKTRLHTEQSVCKTETGIDQQKANDVIEGNIDVEDKKVQLYCECILKNFNILDKNNVFKPQGIKAVMELLIDENSVKQLVSDCSTISEENPHLKASKLVQCVSKYKTMKSVDFL

>AmelOBP4

MKITIVSLLCVIYCALVHADTVAILCSQKAGFDLSDLKSMYESNSEEQMKKLGCFEACVFQKLHFMDGNTLNVEKLESGTRELTPDDFTEDVHEIIEQCVSKAADEDECMVARKYIDCALEKMKFLDDELEKIAGN

>AmelOBP11

MKAAEIWLVSLYWYLILQIALVYGEISDIDEFREMTSKYRKKCIGETKTTIEDVEATEYGEFPEDEKLKCYFNCVLEKFNVMDKKNGKIRYNLLKKVIPEAFKEIGVEMIDSCSNVDSSDKCEKSFMFMKCMYEVNPIAFIAP

>AmelOBP9

MFKNYHFFFILVITLIFLYFGEADIKKDCRKESKVSWAALKKMKAGDMEQDDQNLKCYLKCFMTKHGILDKNAEVDVQKALRHLPRSMQDSTKKLFNKCKSIQNEDPCEKAYQLVKCYVEFHPEVLQTVPFL

>AmelOBP12

MLYNNLTIVIILIMCGVQNLRARSVNIFQDIADCVDRSNMTFHELKKLRDSSEARIKLINEEENFRNYGCFLACIWQQTGVMNGSELSTYNIAGIIEGQYHDDEDLKTFFHKIALTCEDDVHRKFLHVNDECDVALSFKLCMLKAMRNYP

>AmelOBP13

MKTIIFIFAFCLVGILAVSEESINKLRKIESVCAEENGIDLKKADDVKKGIFDKNDEKLACYVDCMLKKVGFVNADTTFNEEKFRERTTKLDSEQVNRLVNNCKDITESNSCKKSSKLLQCFIDNNLMKIFE

>AmelOBP7

MKKFLVIFVYILSVAVIIRANGINEILKIMAVSMKDIRYCIIHMGLTFKDFIKMQELLQEEDISEGNIKKYLTNYSCFITCALEKSHIIQNDEIQLDKLVEMANRKNISIDVKMLSECINANKSTDKCENGLNFIICFSKLLSDMYEDTFEDTLKHKSYV

>AmelOBP21

MKTIVIISAICVCVGALTLEELQIGLRAVIPVCRIDSGIDEKKEDDFRNGIIDVENEKVQLFSECLIKKFNAYDDGGNFNEVVVREIAEIYLDENEVNKLITECSAISDADIHLKSSKLIKCFAKYKTLKEIMNE

>AmelOBP20

MKTIVVIFAFCICVNAMTIEELKIQLHDVQEICKTESGIDQQTVDDINEVNFDVEDEKPQRYNECILKQFNIVDESGNFKENIVQELTSIYLDENVIKKLVAECSVISDANIYIRFNKLVKCFGKYKTMKEVLNL

>AmelOBP19

MKTIVVIFAFCICVNAMTIEELKIQLRDVQEICKAESGIDQQTVDDINEVNFDVEDEKPQRYNECILKQFNIVDESGNFKENIVQELTSIYLDENVIKKLVAECSVISDANIYIRFNKLVKCFGKYKTMKEVLNL

>AmelOBP18

MKTFVIISAICVCVGALTLEEFQIGLRAVVPICRIETSIDQQKEDDFRDGNIDVEDEKVQLFSECLIKKFNGYDDGGNFNEVVIREIAEIFLDENGVNKLITECSAISDADLAVKSAKLLKCIGKYKTLKEMLSG

>AmelOBP16

MKTFVIIFAICVCVGAMTHEELKTGIQTLQPICVGETGTSQKIIDEVYNGNVNVEDENVQSYVECMMKKFNVVDENGNFNEKNTRDIVQAVLDDNETDQLIVECSPISDANVHIKISKIFQCFMKYKTITDILNS

>AmelOBP17

MKTIVIISAICVCVSAMTLDELKSGLHTVQSVCMKEIGTAQQIIDDINEGKINMDDENVLLFIECTMKKFNVVDENANFNEKISSDIVRAVLNDNEADQLLAECSPISDPNALIKISKILECFFKYKTINQILNS

>AmelOBP15

MKTILIISAICICVGALSIKDFQNAIRMGQSICMAKTGINKQIINDVNDGKINIEDENVQLYIECAMKKFSFVDKDGNFNEHVSREIAKIFLNENEINQLITECSAISDTNVHLKITKIFQCITKFKTINDILNS

>AmelOBP3

MKTIVILLFTLCIVSYMMVRCDDITLCLKQENLNLDDIDSLLEDESERMLRKRGCIEACLFHRLALMNDNVFDVSKFDVYLNDTDMDMDLKDSIRKIIRQCVDNAKNEDKCLTAQKFSRCVIDYVKFHITQYMISNANSNTTSEEESSDNST

>AmelOBP8

MTIEELKKTIKNLRKVCSKKNDTPKELLDGQFRGEFPQDERLMCYMKCIMIATKAMKNDVILWDFFVKNARMILLEEYIPRVESVVETCKKEVTSTEGCEVAWQFGKCIYENDKELYLAP

>AmelOBP10

MKYSILLSLLITCLICSPSVHCGTRPSFVSDEMIATAASVVNACQTQTGVATVDIEAVRNGQWPETRQLKCYMYCLWEQFGLVDDKRELSLNGMLTFFQRIPAYRAEVQKAISECKGIAKGDNCEYAYRFNKCYAELSPRTYYLF

>AconOBP1

MNSINVFCLFVLLFRQIDALQCRSGNEQTSDELRKIMEKCNHRQTDGKHDDNSSVDYSSDNSSEEMMFSKDFFTNNKKKTENVTKNSGSTSGIDSSDHYKRQFSNRPYDINYSNYSGLQQTTNTKNSNDDTAKISCNIHCFFDELNLVDQRGFPERISVTKSMIKNIHYSELRDFIEESILECFQFLSNDPNQDKCEYSQNLVNCFADKGKEGCEDWDE

>AconOBP2

MNHHKSINISSIFIAIVIIAAVNIFNIEARMTMAQIQNAMKPAGKTCAGKTGVAKEVLAQTREGIFPEDRDLMCYHACLLKMMKMMTKDNKIAIETMMKQIDLMMPEDLIQRTKDVSQKCYDELTTDEPCEMSWQFVKCYSDTDRSLYFFP

>AconOBP3

MQNILRFLCLVQVVLVACGSRPSFVSDEMIAGAASVVNACQTQTGVATTDIEAVRNGEWPDSRSLKCYMYCLWAQFGLVDNNRELSLNGMLTFFQRMPAYRAEVDRAIRECKGLSKYYANGDDCQYAYTFNQCYAEHSPQTYYLF

>AconOBP4

MNSFTVASLIVLVTLCAGFLPTHAEEEDDDNPLKALAKESIEHCKDKLSDENKELLKKNRKADNREIRCFQACVLNHVGALKDTKFDMDKIKQVVNDNVDEVERDDWIAAINVCREDGEKETDECDVAGAFVQCFNNYEESDEE

>AconOBP5

MSRIIVNCVLLGIILQAGLVPAKRPDFVTDEMVAMIKDDKDRCMQEHGTTEALIERVNDGDIPNDKAITCYMYCLFESFSVIDEDGVLEADMLTGFFPEDIQAKGGPILSACASQDGADNCEKVYNIAKCVHSKMPEMWFMV

>AconOBP6

MKVLYHCSLLLFTVIVLINADNSDPHKPIRFKCCSDLTLTEDVLEAGVSHPEDFGCYLSCFLQNLNIMDDKGVFDPAVATQSVAADLREESKNDIYACYEMRKDEPTDDLCKTAYGMINCFRERSPKLYEMMGIFRAPGK

>AconOBP7

MTKILIVISALSLLALVHAGPIPKEFQDVAAGIRETCMKESSVSLELLERAGKGDFADDETLKCYLKCVFDQFRLISTKGFNYKAFLTLAPPDLKDKAAKLIELCGETTGKPGDMCDLSWNINKCMYNAYPDVYFIF

>AconOBP8

MVILNRTIRMNFLQILIVSLVLIQTNADIRRDCREQTGVSWDALKRLKAADFNQTDHKLKCYLKCFMTMNGIFNEGDVDVERVLRHLPRSLQESSRTTLEYCKKFPSKDACDKAFQLAKCYFKFQPEVLRSVSFV

>AconOBP9

MKSFIAVLLCTFVVGVLSGGPMHEKIVKCKEELGVEEDAIRNMMKNNDYNDPTVRCFNACLMKSFGKMAEDGTVNKDAVSEHVPPHVDREQFIEAATVCMEEKGTDECDTANLIHKCLKDKKVIPSGLPPPPPQ

>AconOBP10

MKTLVVILVVCIVGVFGGLSDEQKEKLRIHRKTCETETGVEKTLVDNAHRGNWAESDPKLRCFAACMLKRMAMMDDSGNFNEAETRKKISSDIPADKVDEVINKCKDMKGADSCETGLKLMKCYNDQRAVIMA

>AconOBP11

MACLLQGLKLVNPDGSLNSQVAIDKIPDTIDSRDIIVNAINVCSQRKGSEPCSTAHELFNCIHENKIPELLLG
