# Supplementary material for: Spatial Expression Analysis of Odorant Binding Proteins in Both Sexes of the Aphid Parasitoid Aphidius gifuensis and Their Ligand Binding Properties
Source: Front Physiol. 2022 May 4;13:877133. doi: 10.3389/fphys.2022.877133 (PMC9115719; doi:10.3389/fphys.2022.877133)
Supplement: Supplementary file 3 [file DataSheet1.docx]

Supplementary Table.1 List of RNA-seq secquencing output data quality

| Sample | Raw Reads | Clean reads | Clean bases | Error (%) | Q20(%) | Q30(%) | GC (%) |
| --- | --- | --- | --- | --- | --- | --- | --- |
| AgifantF_1 | 27586216 | 26482992 | 3.31G | 0.03 | 96.17 | 92.90 | 34.19 |
| AgifantF_2 | 27586216 | 26482992 | 3.31G | 0.03 | 94.85 | 90.83 | 34.23 |
| AgifantM_1 | 25116646 | 24271556 | 3.03G | 0.03 | 95.27 | 91.73 | 31.02 |
| AgifantM_2 | 25116646 | 24271556 | 3.03G | 0.04 | 93.33 | 88.64 | 31.04 |


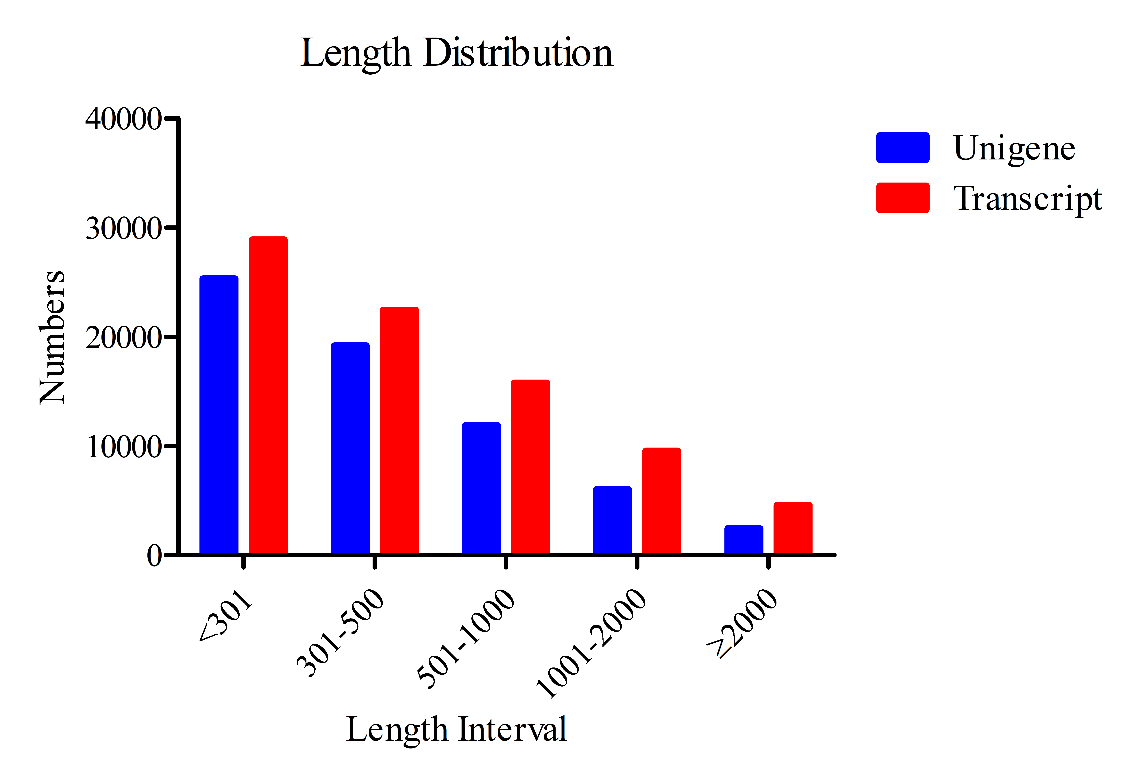


Fig.1 Splice length distribution of unigene and transcript

The abscissa is the length interval of the spliced transcript / unigene, and the ordinate is the number of times the spliced transcript / unigene of each length appears.

Supplementary Table2. Gene annotation success rate statistics

|  | Number of Unigenes | Percentage (%) |
| --- | --- | --- |
| Annotated in NR | 18408 | 27.95 |
| Annotated in NT | 5625 | 8.54 |
| Annotated in KO | 7551 | 11.46 |
| Annotated in SwissProt | 12484 | 18.95 |
| Annotated in PFAM | 15070 | 22.88 |
| Annotated in GO | 15951 | 24.22 |
| Annotated in KOG | 9462 | 14.36 |
| Annotated in all Databases | 2752 | 4.17 |
| Annotated in at least one Database | 22311 | 33.87 |
| Total Unigenes | 65854 | 100 |


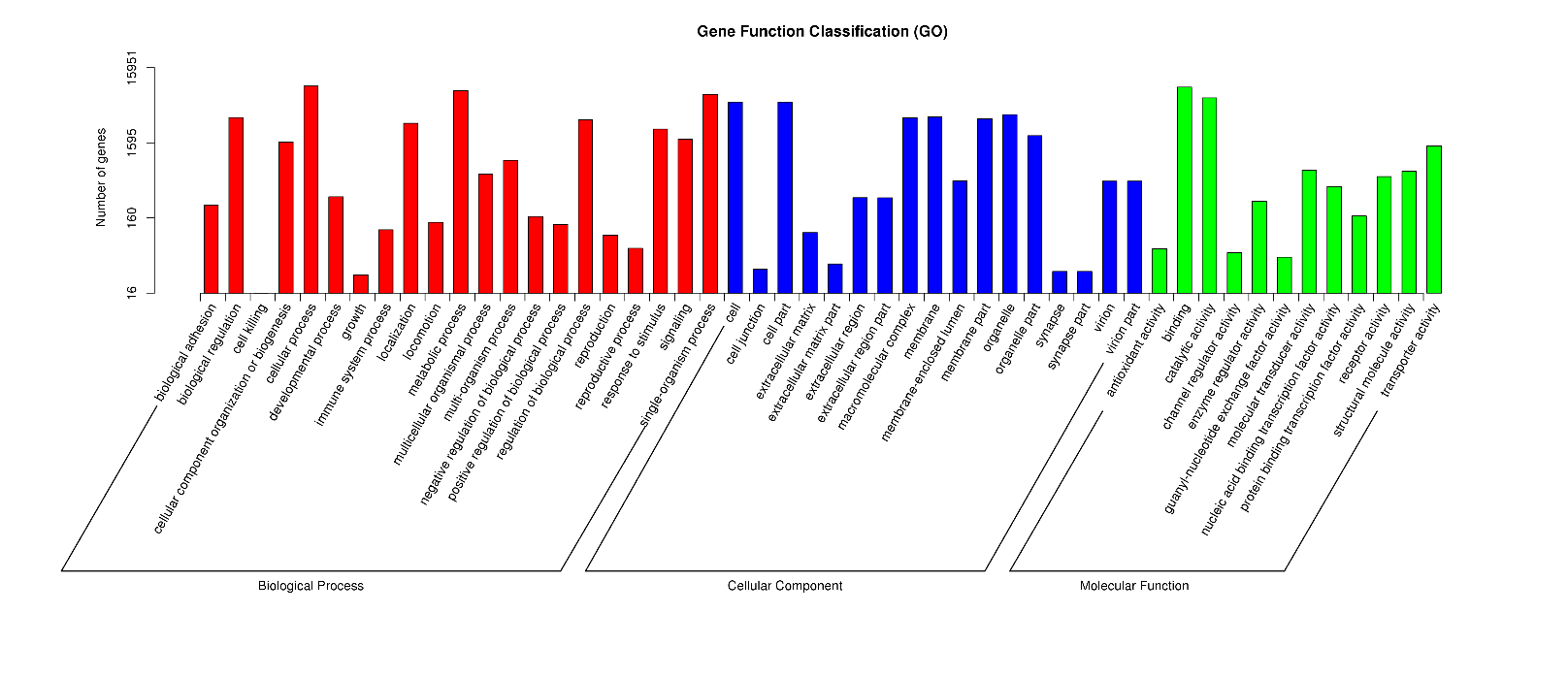
Fig.2 gene function classification

The abscissa is the next GO term of the three major categories of GO, and the ordinate is the number of genes annotated under the term (including subterms of the term). Three different classifications represent the three basic classifications of Go term (from left to right are biological processes, cellular components, and molecular functions)


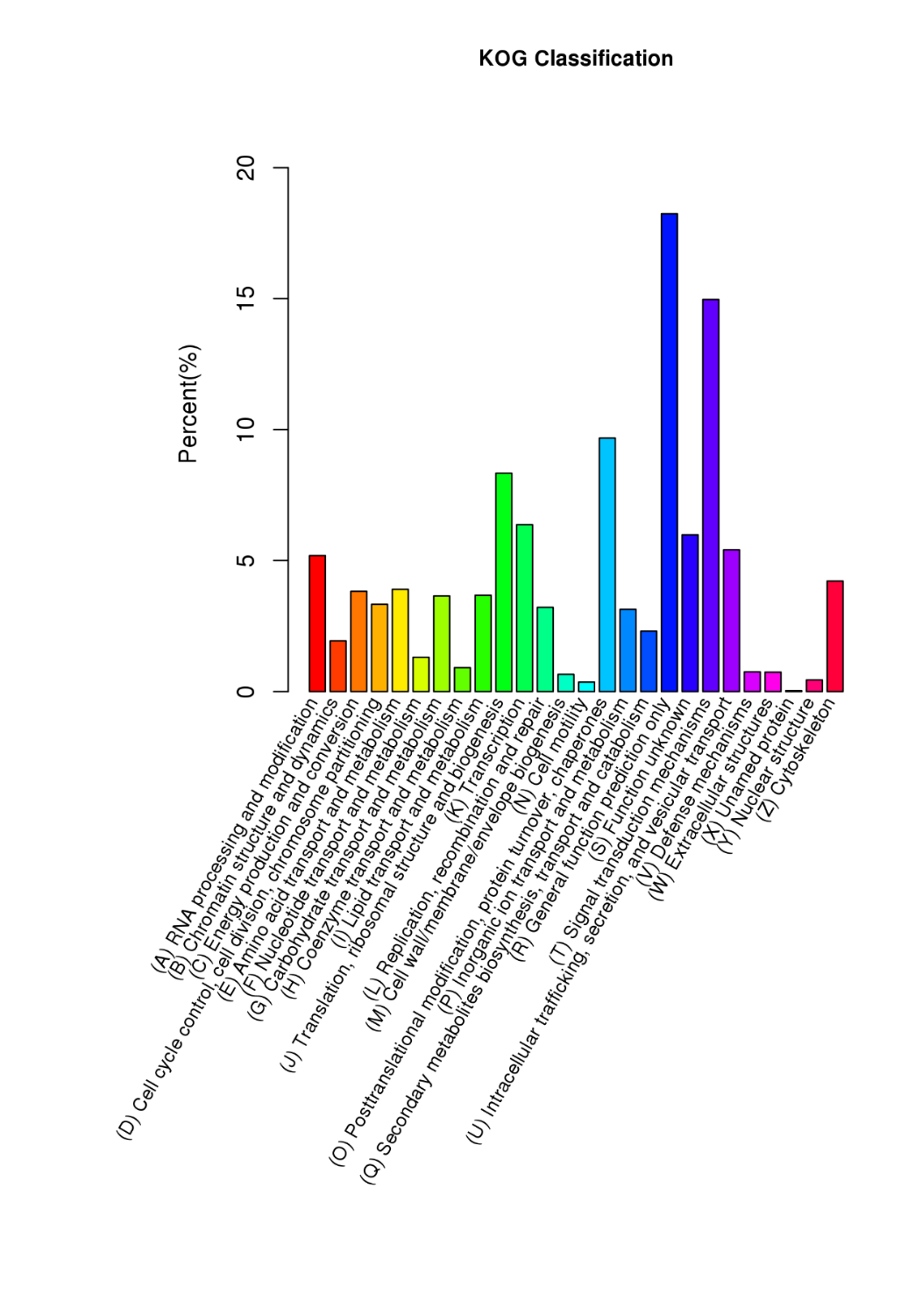


Fig.3 KOG classification

The abscissa is the names of 26 groups of KOG, and the ordinate is the ratio of the number of genes annotated to this group to the total number of genes annotated.


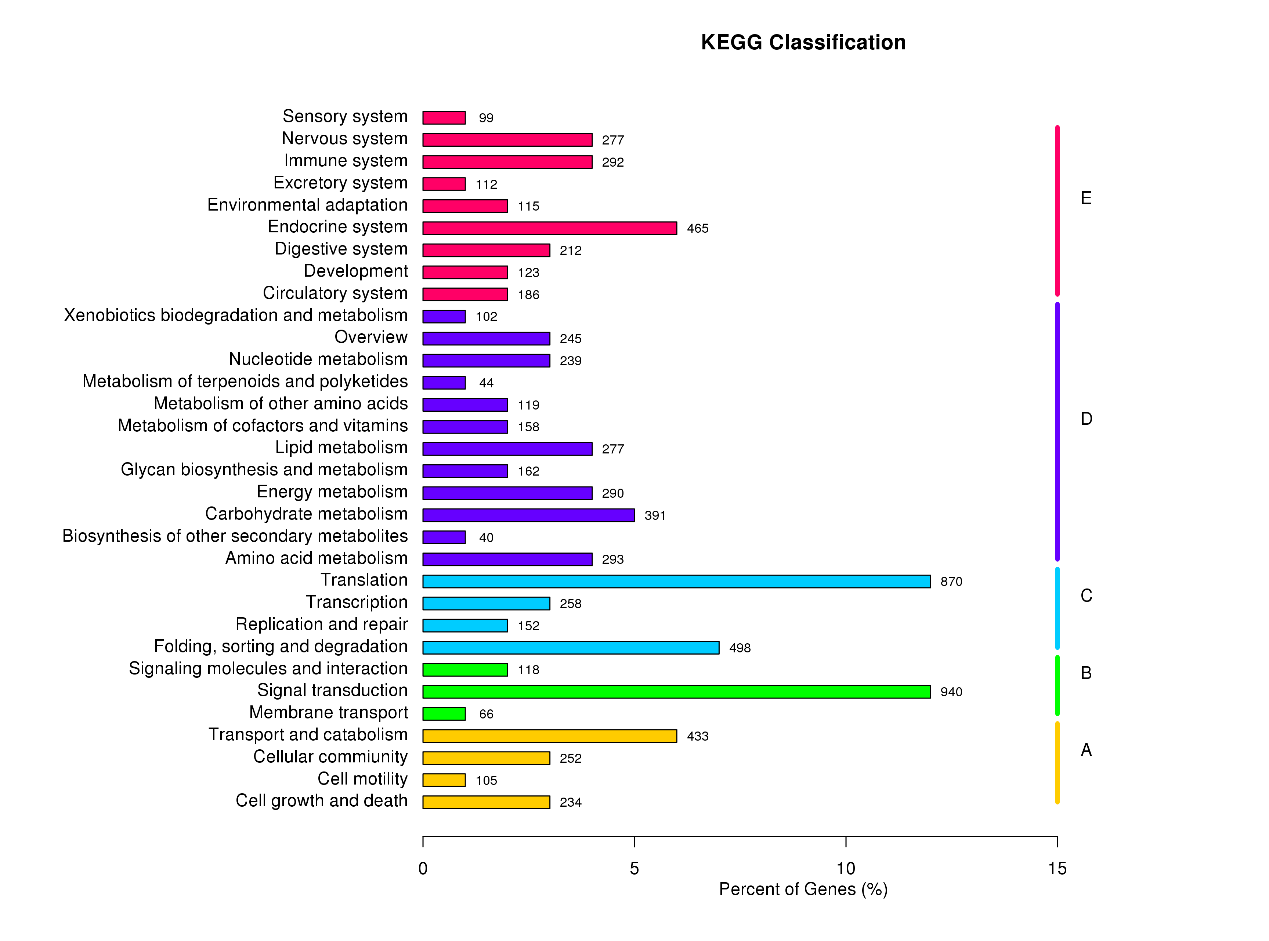


Fig.4 KEGG classification

The ordinate is the name of the KEGG metabolic pathway, and the abscissa is the number of genes annotated under the pathway and their proportion to the total number of genes annotated. Divide genes into five branches according to the KEGG metabolic pathway involved: cellular process (A, Cellular Processes), Environmental Information Processing (B, Environmental Information Processing), Genetic Information Processing (C, Genetic Information Processing), metabolism (D, Metabolism), organic systems (E, Organicismal Systems).


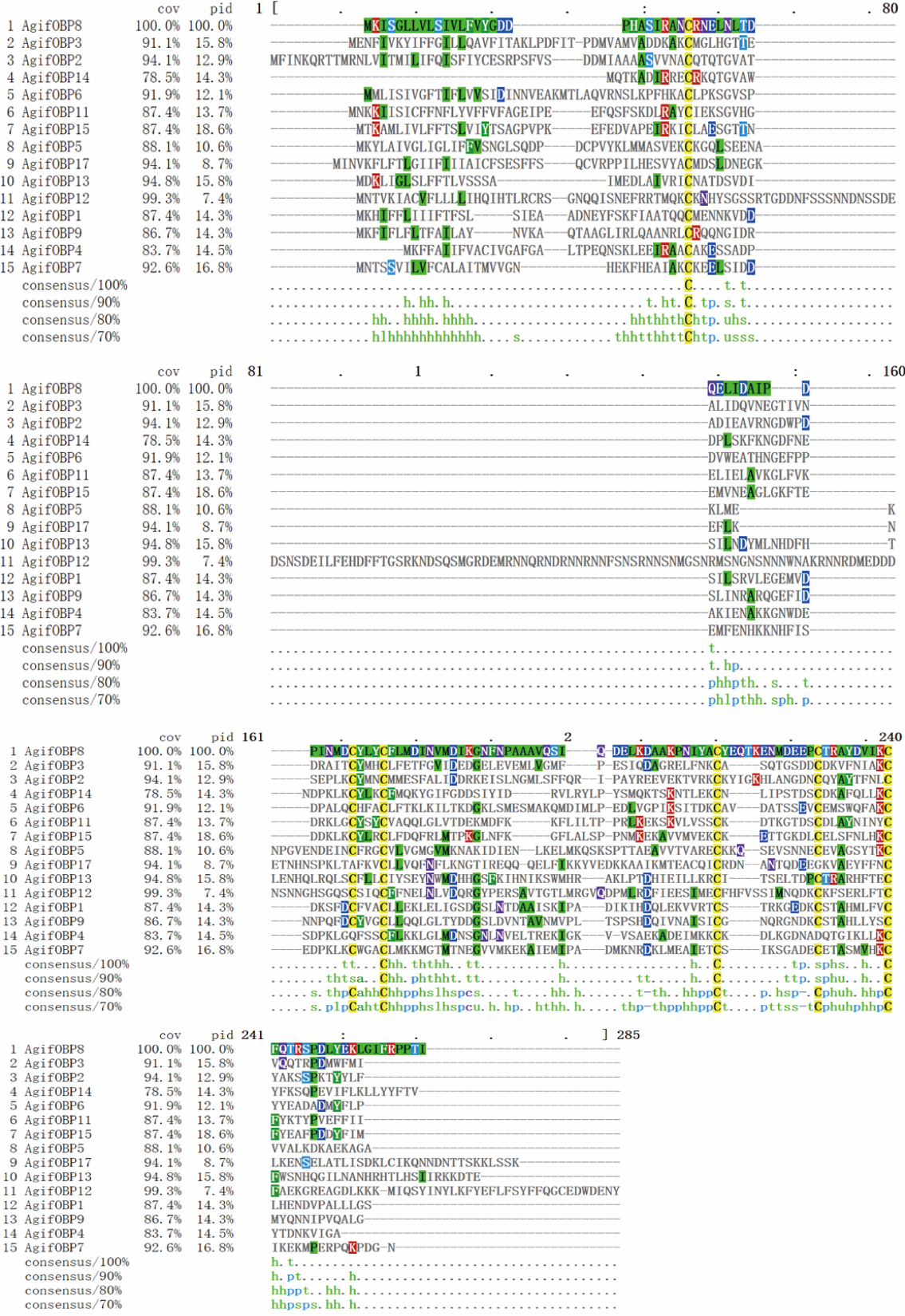


Fig.5 Multiple sequence alignment of 15 odorant binding protein (OBP) genes in *Aphidifus gifuensis*. The conserved cysteine residues are highlighted by red boxes. The conserved Cys residues are indicated, represents conserved sequence identity >75%.

| Gene names | direction | sequences（5'→3'） |
| --- | --- | --- |
